# Abortive Autophagy Induces Endoplasmic Reticulum Stress and Cell Death in Cancer Cells

**DOI:** 10.1371/journal.pone.0039400

**Published:** 2012-06-26

**Authors:** Sofie Claerhout, Bhaskar Dutta, Wouter Bossuyt, Fan Zhang, Catherine Nguyen-Charles, Jennifer B. Dennison, Qinghua Yu, Shuangxing Yu, Gábor Balázsi, Yiling Lu, Gordon B. Mills

**Affiliations:** 1 Department of Systems Biology, The University of Texas MD Anderson Cancer Center, Houston, Texas, United States of America; 2 Department of Biochemistry and Molecular Biology, The University of Texas MD Anderson Cancer Center, Houston, Texas, United States of America; Institut national de la santé et de la recherche médicale - Institut Cochin, France

## Abstract

Autophagic cell death or abortive autophagy has been proposed to eliminate damaged as well as cancer cells, but there remains a critical gap in our knowledge in how this process is regulated. The goal of this study was to identify modulators of the autophagic cell death pathway and elucidate their effects on cellular signaling and function. The result of our siRNA library screenings show that an intact coatomer complex I (COPI) is obligatory for productive autophagy. Depletion of COPI complex members decreased cell survival and impaired productive autophagy which preceded endoplasmic reticulum stress. Further, abortive autophagy provoked by COPI depletion significantly altered growth factor signaling in multiple cancer cell lines. Finally, we show that COPI complex members are overexpressed in an array of cancer cell lines and several types of cancer tissues as compared to normal cell lines or tissues. In cancer tissues, overexpression of COPI members is associated with poor prognosis. Our results demonstrate that the coatomer complex is essential for productive autophagy and cellular survival, and thus inhibition of COPI members may promote cell death of cancer cells when apoptosis is compromised.

## Introduction

Cancer cells encounter several barriers such as pH changes and a restricted supply of nutrients and oxygen during cancer initiation, progression, and dissemination [1]. To reestablish proper cellular function and homeostasis under low to moderate levels of stress, cancer cells activate protective cellular processes such as autophagy. Autophagy is a highly conserved process found from yeast to mammals. It is a unique degradation process characterized by the sequestration of bulk cytoplasm including proteins and organelles, which are delivered to the lysosomal system for final digestion [2]. Removal and degradation of these cellular components produces energy and the building blocks necessary for the survival of cancer cells during metabolic stress. The completion of this process is designated productive autophagy or autophagic flux [2]. However, non-productive, uncontrolled, or prolonged autophagy leads to what has been designated “autophagic cell death” [3], [4] or, as we refer to, “abortive autophagy” [5].

In cancer, the process of autophagy has been proposed to contribute both to tumor suppression and progression. On the one hand, autophagy suppresses tumors by limiting initiation and progression. The expression of Beclin 1, the mammalian homolog of yeast autophagy gene Atg6 which is critical for induction of autophagy, is decreased in many cancer tissues and cancer cell lines [6]. In addition, the ultraviolet radiation resistance–associated gene (*UVRAG*), necessary for the tumor suppressor function of Beclin 1, is also monoallelically mutated in many human cancers [7]. Moreover, autophagy also suppresses tumorigenesis by providing the ATP required for DNA repair [8] or by eliminating the long-lived protein p62 [9]. On the other hand, autophagy may promote tumorigenesis by overcoming the stress evoked by the lack of nutrients and oxygen in pre-invasive and rapidly growing tumors. Our lab has shown that the LKB1-AMPK pathway-dependent phosphorylation of p27 at threonine 198 stabilizes p27 and permits cells to survive growth-factor withdrawal and metabolic stress by inducing autophagy [10]. This autophagic response may contribute to tumor-cell survival during growth-factor deprivation or disrupted nutrient and energy metabolism. Thus, depending on the cellular context and the strength and duration of the stress signals, autophagy either prevents or promotes carcinogenesis.

In addition to its role in carcinogenesis, autophagy also modulates the efficacy of therapeutic drugs. Induction of a “productive” autophagic process by chemotherapy and radiation permits the survival of cancer cells [11]. This suggests that autophagy modulators may sensitize tumor cells to conventional cancer therapies [11], [12]. By targeting key proteins in productive autophagy, the cells may undergo “abortive autophagy”, an alternative death mechanism for cancer cells resistant to apoptosis-inducing agents [11], [13]. Although the process of productive autophagy has been studied extensively, it is unclear which factors regulate abortive autophagy. Identifying these modulators could improve the efficacy of conventional therapy and contribute in the discovery of novel treatments for cancer patients.

The purpose of this study was to identify regulators of abortive autophagy and elucidate their effect on signaling mechanisms and cellular survival in cancer cells. We employed small interfering RNA (siRNA) screens in combination with validation and functional studies. Members of the coatomer complex I (COPI), which are involved in cellular trafficking, emerged as top hits from our screens modulating autophagy and cell death in cancer cells. Abortive autophagy and endoplasmic reticulum (ER) stress was observed after knockdown of COPI components. Additionally, COPI depletion affected phosphoinositide 3-kinase (PI3K)/AKT signaling pathway. Altogether, the effects of COPI on cell death, autophagy and ER stress were more pronounced in cancer cells than in normal cells. This is consistent with the overexpression of COPI proteins and genes seen in cancer cell lines and patient samples.

## Materials and Methods

### Cell Lines and Reagents

The human breast cancer cell lines MDA-MB-231 [10], MDA-MB-468 [10], BT549 (American Type Culture Collection (ATCC); Manassas, VA), T47D [10], SKBR3 (ATCC), HCC1428 (ATCC) and MCF7 [10], the ovarian cancer cell lines SKOV3 (ATCC), and OVCAR3 (ATCC) were maintained in RPMI 1640 (Invitrogen, Carlsbad, CA) and 5% FBS. MDA-MB-231, MDA-MD468, and SKOV3 cells stably transfected with GFP-LC3 were grown in the same medium. The osteosarcoma cell line U2OS (ATCC) and U2OS GFP-LC3 [10] were cultured in DMEM and 10% FBS. MCF10A (ATCC) was cultured in DMEM/F12 and 5% horse serum with 20 ng/ml EGF, 0.5 mg/ml hydrocortisone, 100 ng/ml cholera toxin, and 10 µg/ml insulin. GFP-LC3 stable cell lines were generated as described in [10]. Cell lines were validated by STR DNA fingerprinting using the AmpFℓSTR Identifiler kit according to manufacturer instructions (Applied Biosystems cat 4322288). The STR profiles were compared to known ATCC fingerprints (ATCC.org), to the Cell Line Integrated Molecular Authentication database (CLIMA) version 0.1.200808 [14] and to the MD Anderson fingerprint database. Imatinib, a kind gift from Dr. S. Kondo at MD Anderson Cancer Center, and Bafilomycin A1 (Sigma-Aldrich, St. Louis, MO) were dissolved in DMSO. Brefeldin A (Sigma-Aldrich) and rapamycin (Cell Signaling Technology, Beverly, MA) was dissolved in methanol.

### siRNA Screens

#### Accell siRNA screen for autophagy modulators

MDA-MB-231, U2OS, and SKOV3 cells stably transfected with GFP-LC3 were seeded in 96-well plates at 2500 cells/well for MDA-MB-231 and SKOV3 cells or 1000 cells/well for U2OS cells. After 24 h medium was changed to Accell medium containing 0.05% FBS (80 µl/well) (Thermo Fisher Scientific, Lafayette, CO). Accell siRNAs (G-201000 Human RNAi Global, Lot 08103, in 96-well plates) were dissolved in 10 µl of 1x siRNA buffer and incubated on a shaker at room temperature for 30 min. Accell media (200 µl) containing 0.05% FBS was added to each well and mixed by pipetting five times. The dissolved siRNAs (20 µl) were added to each well to make a final siRNA concentration of 1 µM (100 µl/well). Cells were incubated with siRNA for 48 h. Drugs were diluted to a ten times working concentration and added to each well as indicated (11 µl/well) to make final concentrations of 2 mM 2-Deoxy-D-glucose (2DG), 100 nM rapamycin, and 10 µM imatinib. Cells were washed with PBS and fixed with 4% paraformaldehyde in PBS for 45 min at room temperature. Cells were washed three times with PBS, nuclei stained with Hoechst 33342 for 5 min and washed once again with PBS, and 250 µl PBS was added to the cells. Autophagosome formation was determined by an IN Cell Analyzer 1000 Cellular Imaging and Analysis system (GE Healthcare, Piscataway, New Jersey). Results are shown as % autophagy. In addition, all the genes were combined together for z-score calculation. Z-score of gene *i* was calculated from as *z_i_*  =  (*x_i_* – *μ*)/*σ*, where, *x_i_* is the % autophagy from the autophagy screen, and *µ* and *σ* are the mean and standard deviations based on all genes present in the screen.

#### siRNA screen for viability modulators

We used an arrayed library of 779 siRNA pools (http://www.dharmacon.com/product/Productlandingtemplate.aspx?id=225&tab=1 for gene list) covering the human kinome and kinase related genes (Dharmacon SMARTpool library, Thermo Fisher Scientific, Lafayette, CO) in the MDA-MB-231, MDA-MB-468, SKOV3, and OVCAR3 cell lines. Each pool of synthetic siRNAs was arrayed in 96-well format, and transfections were performed in triplicate plates using 25 nM of siRNA delivered by DharmaFECT I (Dharmacon). Six replicates of RISC-free non-targeting siRNA and siRNA against PLK (toxic control) in each plate provided negative and positive controls for cell death across the entire screen, respectively. Briefly, DharmaFECT I was applied at 0.15 µl/well for 25 nM siRNA delivery in a reverse transfection mode. After three days, the cell viability was assayed with a Cell Titer Blue Cell Viability Assay (Promega, Madison, WI). Cell Titer Blue reagent (resazurin 5 µl/well) was included in the last 3 h incubation. Viable cells reduce resazurin into resorufin, which is highly fluorescent. The fluorescence intensity was measured using a microplate reader at an excitation wavelength of 530 nm and an emission wavelength of 604 nm. Interplate bias was eliminated by normalizing cell viability values by mean centering and scaled to have equal variance and then converted to the corresponding z-scores for each of the three replicate experiments. The z-score of gene *‘i’* for replicate *‘j’* in plate *‘k’* was calculated as *z_ij_*  =  (*x_ij_* – *µ_jk_*)/*σ_jk_*, where, *x_ij_* is the raw intensity value, and *µ_jk_* and *σ_jk_* are the mean and standard deviation of intensities for all samples in plate *k*, respectively. Next, the median z-score was calculated over all replicates of each gene in each cell line, and siRNAs with *z_i_^ave^* values lower than -2 were considered as significantly inhibitory “hits” for the corresponding cell line.

### Transfection and siRNA Reagents

siRNAs (Dharmacon; Thermo Fisher Scientific, Lafayette, CO) were transfected with DharmaFECT I (Dharmacon) or Lipofectamine RNAiMAX (Invitrogen) using the manufacturers’ protocols. The sequences of the siRNAs are as follows: control RISC-free nontargeting siRNA (D-001220-01-05), COPA (L-011835-00), COPB1 (L-017940-01), COPB2 (L-019847-00), COPG1 (L-019138-00), COPG2 (L-019988-01), COPD (L-013063-01), COPE (L-017632-01), COPZ1 (L-020293-01), PLK1 (M-003290-01), BiP1 (L-008198-00-0005), Atg5 (L-004374-00), and Atg12 (L-010212-00).

### Transmission Electron Microscopy

Cell samples were fixed with a solution containing 3% glutaraldehyde plus 2% paraformaldehyde in 0.1 M cacodylate buffer, pH 7.3, for 1 h. After fixation, the samples were washed and treated with 0.1% Millipore-filtered cacodylate buffered tannic acid, postfixed with 1% buffered osmium tetroxide for 30 min, and stained en bloc with 1% Millipore-filtered uranyl acetate. The samples were dehydrated in increasing concentrations of ethanol, infiltrated, and embedded in LX-112 medium. The samples were polymerized in a 70°C oven for 2 days. Ultrathin sections were cut in a Leica Ultracut microtome (Leica, Deerfield, IL), stained with uranyl acetate and lead citrate in a Leica EM stainer, and examined in a JEM 1010 transmission electron microscope (JEOL, USA, Inc., Peabody, MA) at an accelerating voltage of 80 kV. Digital images were obtained using an AMT Imaging System (Advanced Microscopy Techniques Corp, Danvers, MA).

### SDS-PAGE and Western Blot Analysis

Western blotting was performed as previously described [12]. The following antibodies were used: p62 (BD Biosciences, Franklin Lakes, NJ), LC3 (Novus Biologicals, Littleton, CO), IRE1α, BiP, calnexin, Atg5, Atg12, pSer473AKT, pThr308AKT, AKT, pSer235/236 S6, pSer65 4EBP, and 4EBP (all from Cell Signaling Technology, Beverly, MA), COPB1 (Fisher Scientific, Lafayette, CO), and β-actin (Sigma-Aldrich). Antibodies against COPA, COPB2, COPD, COPG1, and COPG2 were a kind gift from Dr. Wieland (University of Heidelberg, Heidelberg, Germany). Quantification of band intensity was performed by Un-Scan-It gel software (version 5.1, Silk Scientific Corporation) and expressed in terms of fold increase of control.

### Confocal and Fluorescence Microscopy

#### Confocal microscopy

Immunostaining for LAMP2 (BD Biosciences; 1/200) or COPB2 (1/1000) was performed using standard procedures. For the tfLC3 assay, cells transiently expressing tfLC3 [15] were grown overnight and treated as described. Cells were fixed with 4% paraformaldehyde and stained with 4′-6-diamidino-2-phenylindole (DAPI). For Annexin-V-Alexa 568 staining, cells stably expressing GFP-LC3 were cultured in eight well chamber slides (BD Biosciences) and treated as indicated. Annexin-V-Alexa 568 (Roche, Mannheim, Germany) staining was performed according to manufacturer’s instructions. All images were taken on an Olympus FV1000 microscope with a 100X lens (N.A. 1.30). Images were acquired and processed using the Fluoview software and ImageJ.

#### Fluorescence microscopy

Immunofluorescence images for p62 (BD Biosciences; 1/200) were acquired using a Nikon Eclipse TE200-E microscope and IPLab imaging software (BioVision Technologies, Exton, PA).

### Crystal Violet Cell Viability Assay

Viability assay (crystal violet) was performed using standard procedures.

### Clonogenic Replating Assay

Cells were grown under various conditions and all cells were collected by trypsinization and were plated at 100–400 cells per well in six-well tissue culture plates. Colonies were grown for 11–14 days in complete growth medium and stained with 0.5% crystal violet solution (80% dH_2_O, 20% methanol, 0.5% crystal violet). Pictures of the colonies were taken using FluorChemE imaging system (Cell Biosciences, Inc.), and the number of colonies was analyzed using AlphaVIEW SA (version 3.2.3.0; Cell Biosciences, Inc.). The experiment was performed in triplicate and repeated two times.

### RPPA Analysis

The RPPA procedures for antibody staining and signal detection were performed as previously described [10], [16].

### Oncomine Data Base

The Bittner multicancer dataset is available in the public domain at http://www.oncomine.org/main/index.jsp [17], and therefore no IRB approval was required. Tissues with differential expression of COPI complex members (P≤10^−6^) were selected.

### Statistics and Data Analysis

All data were analyzed using the GraphPad Prism 5 software (GraphPad Software, Inc., San Diego, CA, USA). Comparisons between the two groups were statistically evaluated by a two-tailed paired t-test. A p-value of less than 0.05 was considered statistically significant. To compare the gene expression levels in benign and cancer groups, a two-sample Student’s t-test was used. We used the Cox proportional hazard regression model for univariate survival analysis. Cutoffs of the high and low expression groups were optimized to achieve the lowest p-value. All statistical analyses, box plots, and Kaplan-Meier survival plots were performed using R Software (http://www.r-project.org).

## Results

### siRNA Screening Identifies Coatomer Complex Members as Modulators of Viability and Autophagy

To identify novel molecular players in the control of autophagic cell death, we performed two types of RNA interference (RNAi) screens to find genes that when knocked down resulted in both decreased cell viability and formation of autophagosomes. We first screened the human kinome because kinases and kinase-related genes are key regulators of complex cellular functions such as autophagy. siRNAs were transfected in four different cell lines: two ovarian cancer cell lines (SKOV3 [*HER2* amplified; *CDKN2A*, *PIK3CA*, and *TP53* mutants] and OVCAR3 [*PIK3R1* and *TP53* mutants]) and two breast cancer cell lines (MDA-MB-231 [*BRAF*, *CDKN2A*, *KRAS*, and *TP53* mutants] and MDA-MB-468 [*EGFR* amplified; *PTEN*, *RB1*, *TP53*, and *SMAD4* mutants]) with different genetic backgrounds. In total, we identified 67 genes, 9% of the kinome, that when knocked down significantly reduced cell viability (z-score less than -2) (Table S1). Of these 67 positive hits, knockdown of 12 genes reduced the viability of at least two of the cell lines tested. These genes were *AKT2*, *CHEK1*, *COPB2*, *PRKAR2B, RPS6KA2* and *WEE1* (in three out of four cell lines) and *AVPR1B*, *CNKSR1*, *DGUOK, INSRR*, *JAK2, TAF1L* (in two out of four cell lines).

To identify genes also involved in the regulation of autophagy in cancer cells, we conducted an image-based small RNAi library (Accell; Table S2) screen with autophagosome formation as an endpoint. MDA-MB-231, SKOV3, and U2OS cancer cell lines were stably transfected with the green fluorescent protein–microtubule associated protein 1 light chain 3 (GFP-LC3) reporter vector [18], [19]. Because LC3 is degraded during the final stages of productive autophagy [20], the persistent accumulation of LC3 in punctate vesicles was used as a marker for impaired or abortive autophagy. Rapamycin, 2-deoxy-D-glucose (2DG), and imatinib were positive controls for autophagosome formation. Non-targeting scrambled siRNAs, used as negative controls, did not result in autophagy (Fig. S1A–C). In all cell lines tested, we observed that depletion of the coatomer protein complex subunit beta 1 (COPB1) caused an accumulation of the LC3-positive speckles that are suggestive of autophagy (Fig. S1A–C; Table S3), indicating that the autophagy mechanism is conserved among different tumor lineages. In conclusion, using siRNA screens we identified COPB2 and COPB1 as modulators of respectively cell viability and autophagy.

### Coatomer Proteins Modulate Cell Viability

**Figure 1:**
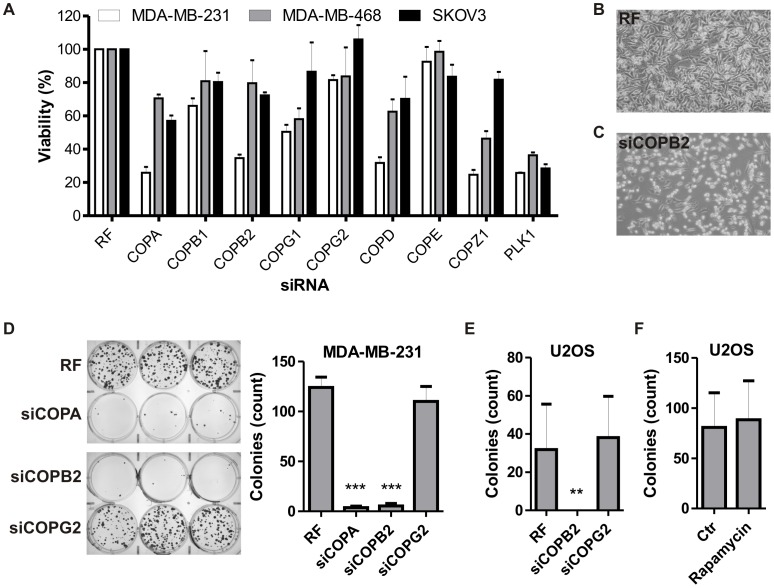
Coatomer proteins modulate cell viability. (A) MDA-MB-231, MDA-MB-468, and SKOV3 cells were transfected with siRNA targeting independent members of COPI. Knockdown of PLK1 was used as positive control for cell death. Cell number after 72 h was measured by crystal violet staining. (B,C) Representative light microscopy pictures of MDA-MB-231 cells treated with RF or siRNA against COPB2. (D) Clonogenic replating efficiency of MDA-MB-231 breast cancer cells after decreased expression of COPA, COPB2 or COPG2 compared to cells treated with risc-free (RF). Results are the mean ± SD from triplicates of two independent experiments. Clonogenic replating efficiency of U2OS cancer cells after treatment with siRNA against COPB2 or COPG2 (E) or rapamycin (F). Results are the mean ± SD from triplicates of two independent experiments. **, p<0.01; ***, p<0.001.

COPB1 and COPB2, components of the COPI complex, were considered as attractive candidates for further study based on the significant impact in cancer cells on both cell viability and autophagosome formation (Table S1 and S3, Fig. S1). In a secondary screen, we validated and analyzed the effects on cell growth and autophagosome formation after knocking down independent members of this complex in three cancer cell lines (MDA-MB-231, MDA-MB-468 and SKOV3). As shown in Figure S2, siRNAs reduced expression of several COPI components. Consistent with our findings from the siRNA screen, depletion of COPA, COPB1, COPB2, COPG1, COPD, and COPZ1 reduced cell growth (Fig. 1A and C). In contrast, cell viability was unaffected in cells treated with non-targeting siRNA (Fig. 1A and B) or only modestly affected in MCF10A, a normal-like breast cell line, after COPB2 knockdown (Fig. S9B). Adding the pan-caspase inhibitor zVAD did not rescue cell death (data not shown) indicating that cell death was caspase-independent. To confirm that cancer cells are committed to death when COPI is impaired, we determined clonogenic recovery after 72 h of siRNA treatment in MDA-MB-231 (Fig. 1D) and U2OS (Fig. 1E) cancer cells. In both cell lines, replating efficiencies were not reduced after COPG2 depletion compared to control, confirming our results. In contrast, reducing expression of COPA or COPB2 almost completely abolished clonogenic recovery (Fig. 1D and E), which is consistent with our viability results (Fig. 1A). Notably, replating efficiency was not reduced in U2OS cells treated with rapamycin (Fig. 1F), consistent with the ability of rapamycin to induce productive autophagy as a survival process. Cell death after COPI knockdown was further visualized and confirmed using Annexin V staining (Fig. S3). Altogether, these results show that most COPI components modulate viability of cancer cells.

### Coatomer Proteins Modulate Autophagy

**Figure 2:**
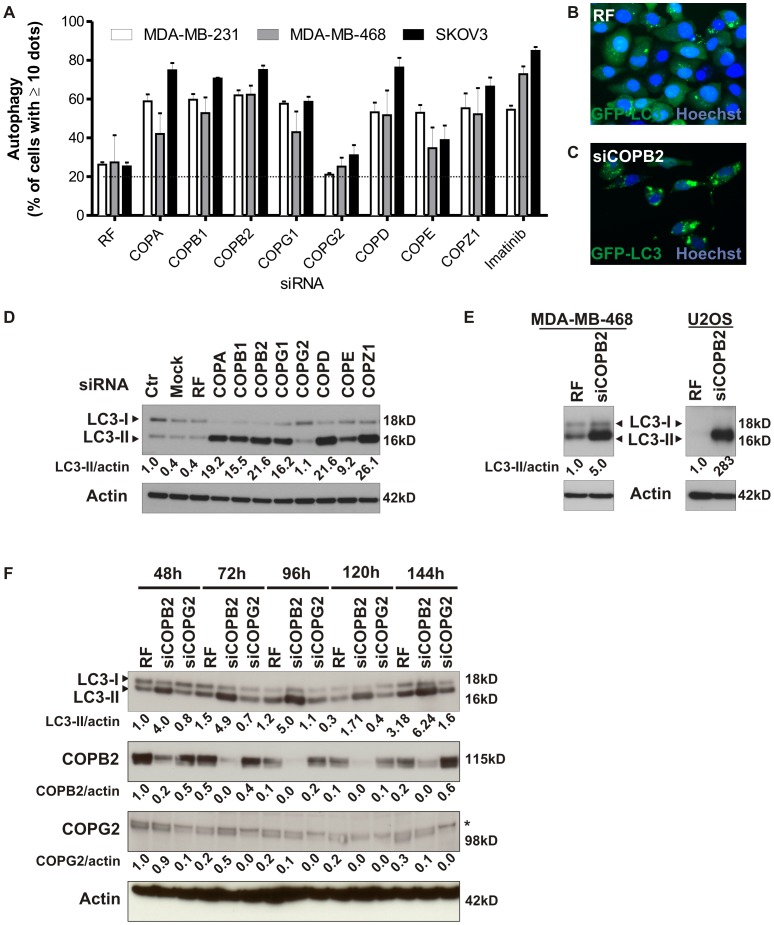
Coatomer proteins modulate autophagy. (A) MDA-MB-231, MDA-MB-468, and SKOV3 cells stably overexpressing GFP-LC3 were transfected with siRNA targeting independent members of COPI for 72 h, and autophagosome formation was analyzed using the IN Cell analyzer 1000 system. Imatinib was used as a positive control for autophagosome formation. Results are the mean ± SD from triplicates. Representative graph of two independent experiments is shown. (B,C) Representative epifluorescent microscopy images (IN Cell Analyzer 1000) of GFP-LC3–positive vesicles in control MDA-MB-231 cells or after COPB2 depletion. (D) Immunoblot analysis of LC3 in MDA-MB-231 cells 72 h after siRNA mediated depletion of independent members of COPI. β-actin was used as a loading control. (E) MDA-MB-468 and U2OS cells treated with RF or COPB2 siRNA were assayed for protein levels of LC3. (F) MDA-MB-231 cells were transfected with siRNA against COPB2, COPG2 or risc-free (RF) and grown for the indicated times, lysed, and immunoblotted with antibodies to LC3, COPB2, COPG2 and β-actin. *, aspecific band.

**Figure 3:**
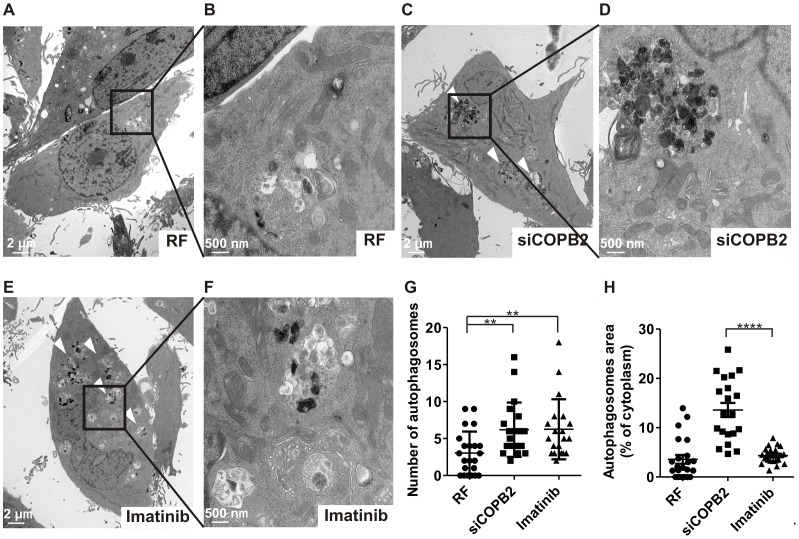
COPI depletion increases autophagosome formation. (A–F) Transmission electron microscopy analysis of autophagy in MDA-MB-231 cells treated with RF or siRNA against COPB2 for 48 h or imatinib for 24 h. White arrowheads indicating autophagosomes are shown in panels C and E. (B, D, F) High magnification images of boxed areas. Scale bars indicate 2 µm (A, C, E) and 500 nm (B, D, F). (G) Number of autophagosomes per cell was calculated by counting the number of double membrane organelles in 20 or more individual cells. (H) Percentage autophagosomal area was calculated by measuring area covered by double membrane organelles in the cytoplasm of 20 or more individual cells. **, p<0.01; ****, p<0.0001,

Next, we tested whether depletion of different independent COPI complex proteins initiated autophagy, using punctate GFP-LC3 formation as a readout. Reduced expression of COPA, COPB1, COPB2, COPG1, COPD, and COPZ1 induced the punctate GFP-LC3 formation in MDA-MB-231, MDA-MB-468 and SKOV3 cancer cell lines (Fig. 2A). These results were comparable to those seen when cells were treated with imatinib, a positive control for autophagosome induction (Fig. 2A–C). Autophagosome formation was also assessed by the conversion of endogenous LC3-I to LC3-II using immunoblotting because the amount of LC3-II correlates with the amount of autophagic membranes labeled with LC3-II [19]. Consistent with the data obtained in cancer cells exogenously overexpressing GFP-LC3 (Fig. 2A–C) and with the exception of COPG2, reducing the expression of the coatomer subunits led to an accumulation of endogenous LC3-II in MDA-MB-231 cells (Fig. 2D). Similar results were found in MDA-MB-468 and U2OS cancer cells (Fig. 2E). We further validated the persistence of the LC3-II increase after COPI depletion by an extended time course analysis (Fig. 2F). A sustained reduced expression of COPI maintained LC3-II expression without apparent degradation of LC3-II, suggestive of impaired autophagy. Electron microscopy analysis (Fig. 3A, C and E; inserts Fig. 3B, D and F) confirmed the formation and marked increase (Fig. 3G) of autophagosomes after COPB2 depletion (Fig. 3C and D) and imatinib treatment (Fig. 3E and F) at the ultrastructural level by the presence of double membrane organelles containing undigested cytoplasmic contents [18]. In contrast, control siRNA (RF) (Fig. 3A and B) did not promote autophagosome formation. Although the increase in autophagosomes was similar between MDA-MB-231 cells treated with COPB2 siRNA and imatinib, we found that autophagosomes induced by COPB2 siRNA were significantly larger compared to autophagosomes induced by imatinib as assessed by quantification of the autophagosomal/cytoplasm area on electron microscopy pictures (Fig. 3H). This observation was further confirmed by determination of the size of GFP-LC3 positive organelles of MDA-MB-231 cells treated with COPB2 siRNA or control siRNA (Fig. S4A). The size of autophagosomes induced by siCOPB2 is also larger than that of starvation-induced autophagosomes (Fig. S4B and S4C). Collectively, these results indicate that COPI depletion results in accumulation of LC3-positive speckles in cancer cells.

### Disruption of COPI Induces Abortive Autophagy

**Figure 4:**
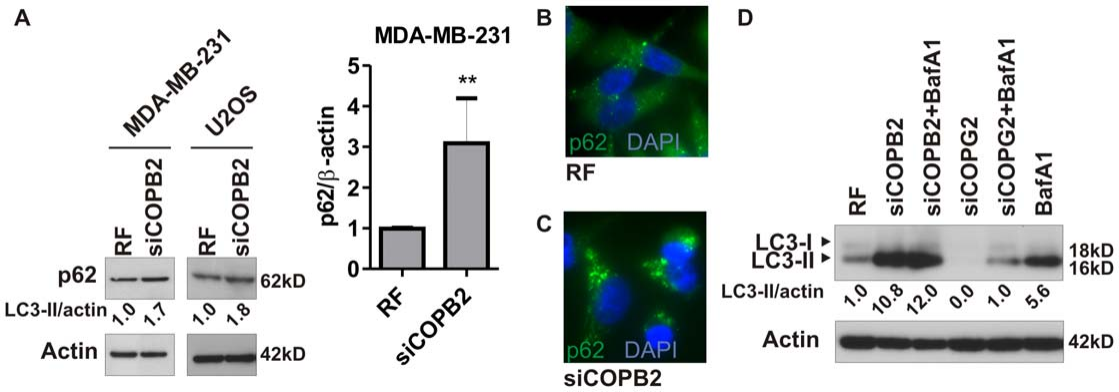
COPI depletion induces abortive autophagy. (A) Indicated cancer cell lines were treated with control siRNA (RF) or COPB2 siRNA for 72 h and p62 level was analyzed by immunoblotting. Quantification of p62 protein levels is shown as the mean ± SD from four independent experiments. **, p<0.01. (B,C) MDA-MB-231 cells were treated as in (A) and p62 was analyzed by immunofluorescence microscopy. (D) COPB2 or COPG2 depleted MDA-MB-231 cells were analyzed for LC3 accumulation in the absence or presence of 50 nM BafA1.

**Figure 5:**
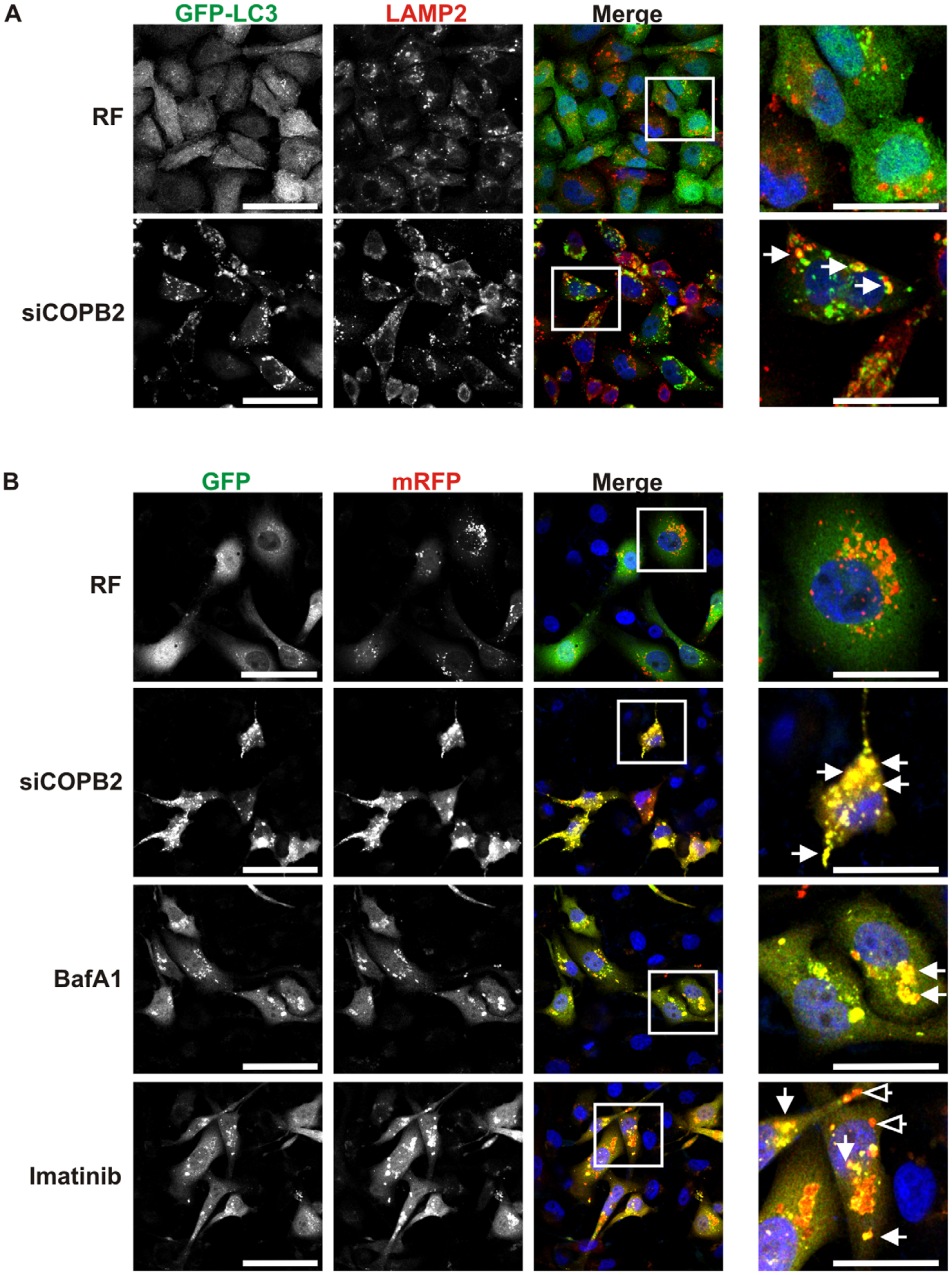
COPI depletion inhibits complete degradation of autophagosomes. (A) GFP-LC3-expressing MDA-MB-231 cells were transfected with risc free (RF) control or with siRNA against COPB2 for 72 h, processed to assess the colocalization between GFP-LC3 and LAMP2 by confocal microscopy. Arrow, colocalization of GFP-LC3 and LAMP2. (B) tfLC3-expressing MDA-MB-231 cells were treated with control siRNA (RF) or COPB2 siRNA for 72 h and BafA1 (50 nM) or imatinib (10 µM) for 24 h. Colocalization of GFP and mRFP was assessed by confocal microscopy. Arrow, RFP-positive/GFP-positive puncta (autophagosome); arrow with open arrowhead: RFP-positive/GFP-negative puncta (autolysosome). Scale bar first column: 50 µM; scale bar last column: 25 µM.

Because depletion of COPI complex members caused cell death and autophagosome formation, we set out to test whether abortive autophagy was the mechanism. To differentiate between abortive and productive autophagy we analyzed p62, which is preferentially degraded during autophagy, but levels remain constant or increased upon the induction of abortive autophagy [21]. As determined by immunoblot analysis of different cancer cell lines (Fig. 4A) and immunofluorescence analysis in MDA-MB-231 cells (Fig. 4B,C), siCOPB2 did not reduce p62 levels, as would be expected of productive autophagy, but increased the levels of p62 as compared to the control levels. In contrast, p62 levels were decreased in MCF10A (Fig. S9D), although COPB2 protein levels were significantly decreased (Fig. S9C). In addition, we used Bafilomycin A1 (BafA1), a lysosome-specific inhibitor of protein degradation and the end stages of the autophagy process to inhibit autophagic flux [18], [22]. BafA1 treatment has been shown to increase LC3-II levels in cells undergoing productive autophagy but has limited effect on LC3-II levels in cells undergoing abortive autophagy [20]. In MDA-MB-231 cells with knockdown of COPB2, BafA1 treatment did not further increase the LC3-II levels (Fig. 4D) in contrast to COPG2 depleted or control cells, indicating impaired degradation of the autophagosomal content by the lysosomes after COPB2 depletion. We confirmed this observation in U2OS cancer cells treated with either BafA1 or rapamycin (Fig. S5). Impaired degradation of the inner luminal content of autophagosomes by lysosomes can result from: 1) failed autophagosome/lysosome fusion; 2) impaired degradation; or 3) incomplete recycling of the autolysosomal components [18], [23]. To determine which of these possibilities was responsible for the increase in LC3-II and p62 in cells with COPI depletion, we first analyzed fusion of autophagosomes (GFP-LC3) and lysosomes (LAMP2) after knockdown of COPI in MDA-MB-231 cells stably transfected with GFP-LC3 (Fig. 5A). Confocal analysis revealed colocalization of GFP-LC3 and LAMP2 in cancer cells treated with siRNA against COPB2 (Fig. 5A), suggesting fusion was not completely blocked by depletion of COPI. We further tested the effect of COPB2 siRNA on autophagosomal degradation using mRFP-GFP tandem fluorescent tagged LC3 (tfLC3; [15]). In cells transiently transfected with tfLC3, GFP−/RFP-positive puncta represent autophagosomes not fused to lysosomes, whereas RFP-positive/GFP-negative puncta represent autolysosomes as GFP is more rapidly quenched by low lysosomal pH [15]. MDA-MB-231 cells transiently transfected with tfLC3 showed colocalization of the mRFP and GFP signal after COPI depletion or BafA1 treatment (Fig. 5B, row 2 and 3), indicative of impaired maturation of the autophagosomes. Treatment of MDA-MB-231 cells with imatinib resulted in RFP-positive/GFP-negative puncta representing the presence of autolysosomes and thus autophagic flux (Fig. 5B, last row). Similar results were obtained in U2OS cancer cells (Fig. S6). In addition, COPB2 depleted cells showed large LAMP2 positive organelles (Fig. S4D). Together, these results indicate that disruption of COPI induces impaired, abortive autophagy possibly through a failure in the maturation and recycling process.

### Abortive Autophagy Induces Endoplasmic Reticulum Stress

**Figure 6:**
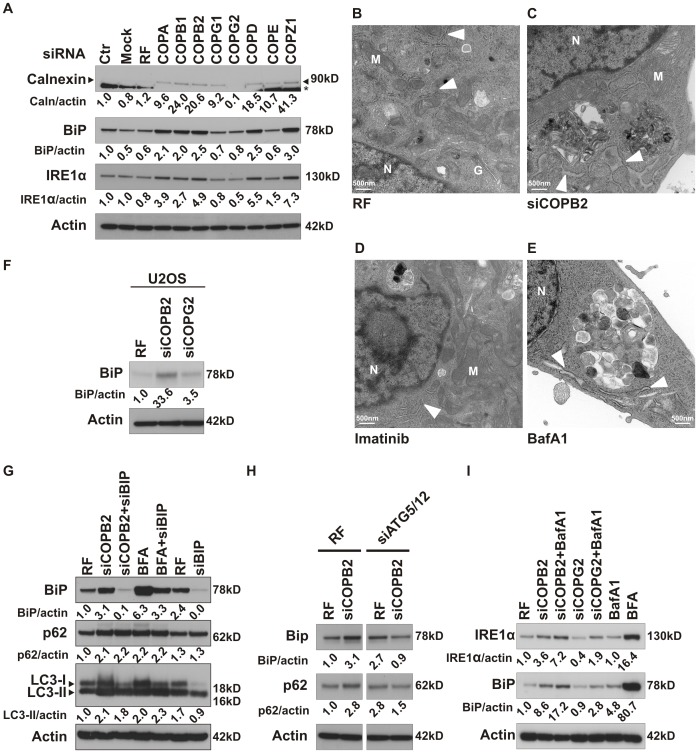
Loss of coatomer subunits promotes ER stress and activates UPR. (A) Independent COPI subunits were depleted in MDA-MB-231 cells and levels of calnexin, BiP, and Ire1α were measured by westernblot after 72 h. *, a non-specific band. (B–E) Transmission electron microscopy analysis of MDA-MB-231 cells after 48 h treatment with non-targeting siRNA (RF) (B), siCOPB2 (C) or overnight treatment with imatinib (10 µM) (D) or BafA1 (50 nM) (E). Electron micrograph showing the dilated ER (white arrow heads in C and E). M: mitochondria, N: nucleus and G: Golgi apparatus. (F) COPB2 or COPG2 depleted U2OS cells were analyzed for BiP levels. (G) LC3, p62 and BiP levels were analyzed by immunoblot after depletion of COPB2 and BFA treatment with or without BiP siRNA. (H) MDA-MB-231 cells were incubated with control siRNA or COPB2 siRNA, either alone or in combination with siRNA targeting the Atg5/Atg12 complex. Images from different parts of one gel were grouped. (I) MDA-MB-231 cancer cells were depleted of COPB2 or COPG2, either alone or with BafA1, and Ire1α and BiP levels were analyzed by immunoblot. BFA was used as a positive control for ER stress.

Coatomer proteins are known to function as carriers between the endoplasmic reticulum (ER) and the Golgi apparatus to return essential proteins to the ER [24]. Therefore, we hypothesized that knockdown of COPI complex proteins would promote ER stress and an unfolded protein response (UPR). Although the UPR is generally viewed as a cytoprotective response, similar to autophagy, prolonged ER stress has been proposed to provoke cell death through different pathways [25]. Compatible with induction of ER stress and activation of UPR, knockdown of independent members of COPI increased calnexin, BiP, and IRE1α levels in MDA-MB-231 cells (Fig. 6A). Ultrastructural analysis (Fig. 6B–E) further confirmed the presence of ER stress. As compared to filament-like ER structures seen in control cells (Fig. 6B) or after imatinib treatment (Fig. 6D), knockdown of COPB2 (Fig. 6C) or BafA1 treatment (Fig. 6E) promoted swelling of ER, a pathognomonic sign of ER stress [26]. ER stress induced by depletion of COPB2 was also observed in U2OS cells (Fig. 6F).

To investigate whether a link exists between ER stress and autophagy, we investigated siRNA-mediated knockdown of BiP, which inhibits most signaling pathways of the UPR [27]. Reduction of ER stress by BiP knockdown slightly decreased LC3-II levels, but not p62 levels, caused by knockdown of COPB2 (Fig. 6G). However, BiP knockdown blocked ER stress caused by brefeldin A (BFA), but we could not detect changes in LC3-II or p62 (Fig. 6G). BFA, an ER stress inducer was used as a positive control not only because it induces the UPR, but also because it inhibits the budding of non-clathrin coated vesicles from the Golgi, effects which are similar to those seen when the COPI complex is depleted [28]. These results suggest that abortive autophagy induced by disrupting the COPI complex is upstream of ER stress. In addition, we found that inhibition of autophagy initiation by depleting ATG5/12 suppressed the COPB2-induced increase in ER stress, as seen by suppression of BiP upregulation (Fig. 6H). Depletion of ATG5/12 also reversed the elevated p62 levels induced by COPB2 siRNA, suggesting that the early stages of autophagy were inhibited (Fig. 6H). Moreover, BafA1 increased BiP and IRE1α protein expression levels in control cells or after COPG2 depletion (Fig. 6I). These data indicate that abortive autophagy provoked by COPI depletion acts upstream of ER stress and that abortive autophagy is sufficient to induce ER stress.

### Abortive Autophagy Decreases PI3K/AKT Signaling

**Figure 7:**
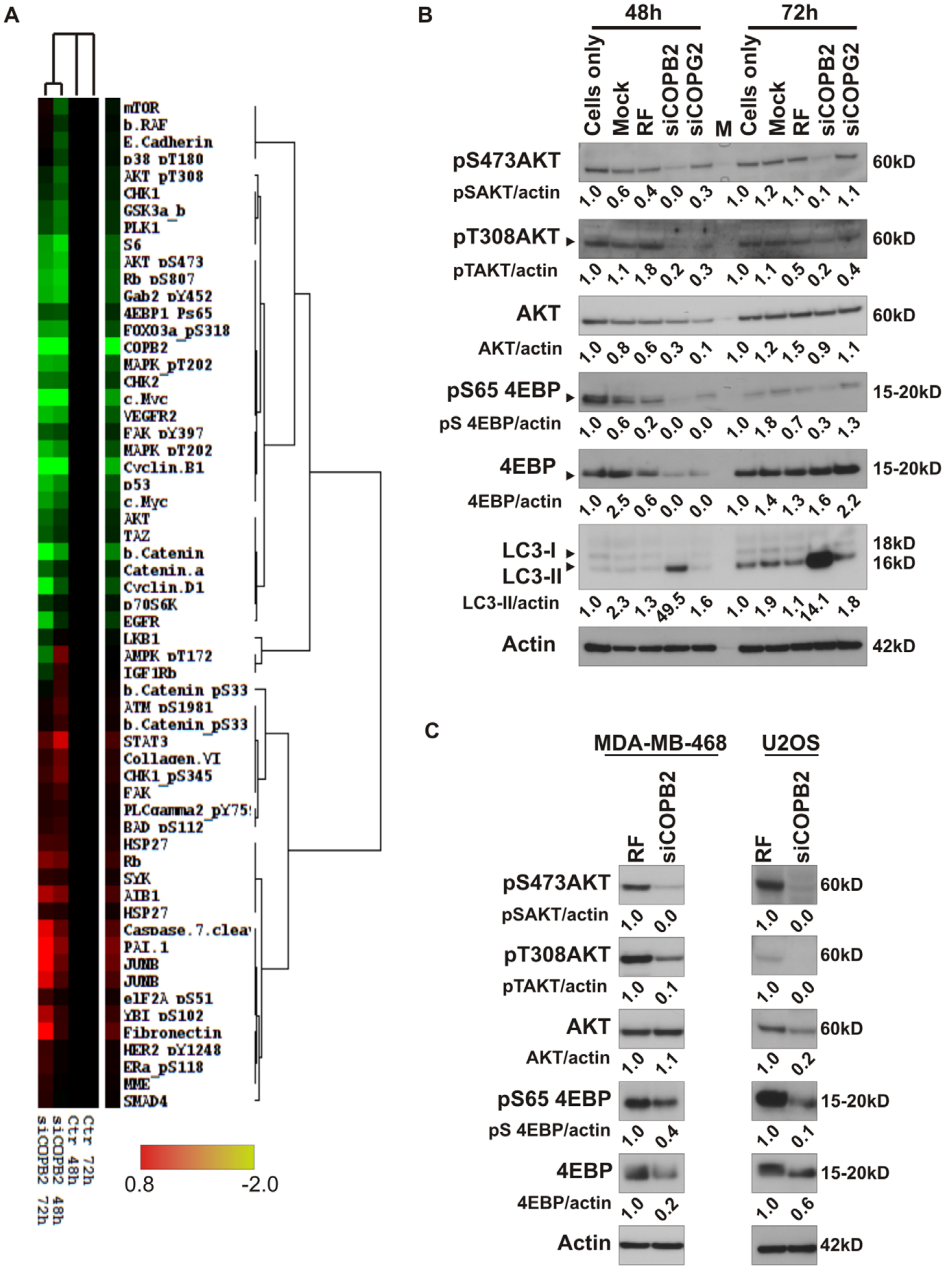
RPPA analysis identifies signaling pathways altered during abortive autophagy. (A) RPPA analysis was performed on lysates of MDA-MB-231 cells depleted from COPB2 for 48 h or 72 h. The heatmap represents values of triplicates normalized to a pool of controls (untransfected, mock transfected and RF transfected cells). Significant changes in proteins related to depletion of COPG2 were excluded. (B,C) Phosphorylated and total levels of the indicated proteins were validated in MDA-MB-231, MDA-MB-468 and U2OS cells. Actin was used as a loading control. M: marker.

To elucidate the mechanisms underlying abortive autophagy generated by COPB2 depletion, we employed a functional proteomic approach, reverse phase protein array (RPPA) to identify signaling molecules that are changed after COPB2 depletion [16], [29]. We quantitatively analyzed the expression profiles of 160 proteins in MDA-MB-231 breast cancer cells 48 or 72 hours post-infection with non-targeting control siRNA or siRNA targeting COPB2 or COPG2. To identify the effect on downstream signaling exclusively associated with COPB2 depletion, we tested for significant differences (p<0.05) between cells treated with siRNA against COPB2 compared to cells treated with non-targeting or COPG2 siRNA. Figure 7A shows the heatmap of 59 proteins whose expression is altered specifically and significantly by COPB2 siRNA. We can distinguish three different groups of protein expression patterns: gradually upregulated, transient expression or downregulated. The RPPA results showed that impaired autophagy induced by siCOPB2 was accompanied by a significant decrease in multiple components of the PI3K/AKT signaling pathway (e.g. pS473AKT, pS65 4EBP, cyclin D1, pY452GAB2) (Fig. 7A). The changes in phospho-AKT expression levels and downstream targets were validated and confirmed by western blot analysis in MDA-MB-231, MDA-MB-468 and U2OS cancer cell lines (Fig. 7B–C). In agreement with these results, chemical induction of abortive autophagy by BafA1 decreased AKT signaling in a panel of breast cancer cell lines (Fig. S7). These results suggest that COPI regulates PI3K/AKT signaling.

### COPI Proteins are Overexpressed in Cancer Cell Lines and Implicated in Cancer Pathophysiology

**Figure 8:**
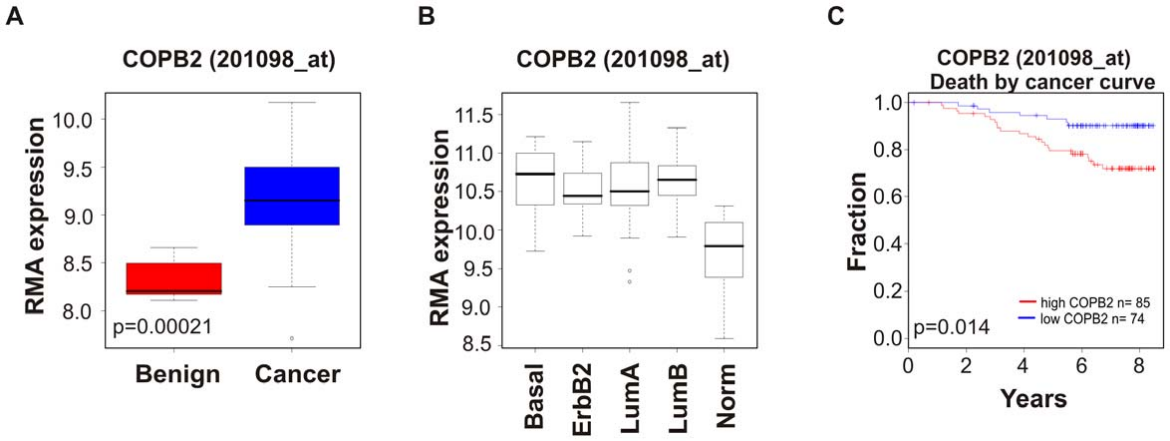
COPI members are overexpressed in cancer. Expression level of COPB2 in (A) ovarian cancer tissue compared with benign tissue and (B) different subtypes of breast cancer. (C) Survival fraction related to high or low COPB2 levels. The cutoff for high and low COPB2 expression is 9.58 (RMA preprocessed log value); this cutoff is optimized to achieve the lowest p value. RMA, Robust Multi-array Average.

Although the role of the COPI complex in trafficking between the ER and Golgi apparatus during recycling and cisternal maturation [30], [31] has been extensively investigated, little is known about the expression and function of the different members of the COPI complex in cancer. In addition, their role in productive autophagy makes them potential oncogenes. To investigate the expression pattern of COPI complex subunits, we analyzed mRNA levels in tissues from 14 different types of cancers using Oncomine. COPI members showed aberrant expression in 6 out of 14 cancer types in the Bittner multicancer dataset (GSE2109; Table 1). These data were independently validated by examination of datasets that compare benign ovarian samples to ovarian cancer samples (Fig. 8A, [32]; Fig. S8, [33]) and different subtypes of breast cancer (Fig. 8B, [34]). High levels of COPB2 correlated with significantly lower survival rates in breast cancer patients (Fig. 8C, [35]). We also detected higher protein levels of COPI components in an array of breast cancer cell lines compared to MCF10A, a non-transformed mammary epithelial line (Fig. S9A).

**Table 1:** *COP*s are overexpressed and implicated in the pathophysiology of a number of cancer lineages.

Tumor Type	*COPA*	*COPB1*	*COPB2*	*COPG1*	*COPG2*	*COPD*	*COPE*	*COPZ1*	*COPZ2*
Bladder	10^−7^								
Pancreatic									
Cervical									
Breast	10^−11^		10^−9^		10^−9^				10^−20^
Liver									
HN									
Lymphoma									
Prostate		10^−9^	10^−7^	10^−6^					
Sarcoma									10^−11^
Lung									
Brain, CNS									
Colorectal			10^−17^			10^−10^	10^−8^	10^−13^	
Ovarian			10^−8^		10^−15^				
Kidney									

Aberrations with P≤10^–6^ are indicated. Data are derived from a reanalysis of Bittner Multicancer Dataset (www.oncomine.org). HN, Head and Neck; CNS, Central Nervous System.

## Discussion

**Figure 9:**
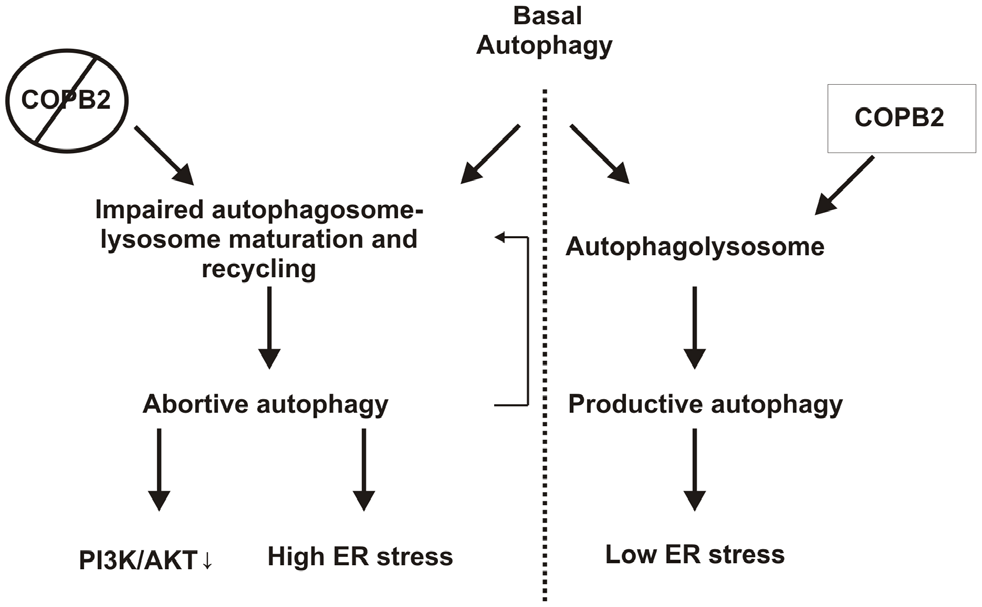
Schematic representation of the role of COPI in the regulation of productive and abortive autophagy. COPI is required for productive autophagy under basal conditions. Depletion of the COPI complex results in abortive autophagy, ER stress and decreased PI3K/AKT signaling.

In this study, we found that members of COPI are critical inhibitors of productive autophagy and cell death in cancer cells. Indeed, depletion of COPB2 was found to induce abortive autophagy or autophagic cell death, which occurred before ER stress and cell death (Fig. 9). In addition, disruption of the COPI complex affected multiple signaling pathways involved in cancer cell survival, most importantly AKT signaling pathway. Moreover, different tumor lineages show higher expression levels of COPI members compared to normal tissue which correlated with worse patient outcome.

COPI is a heptameric coat complex consisting of two subcomplexes, F (COPB1, COPG, COPD, and COPZ) and B (COPA, COPB2, and COPE), which are both required for the binding of membrane proteins [36]. COPI functions in the retrograde transport of luminal and membrane proteins in the ER-Golgi segment of the secretory pathway, by bringing escaped ER resident proteins back to the ER [37] and in the transport of cargo from the Golgi apparatus [38]. The COPI subunits are recruited to the Golgi and assemble into the COPI vesicle coat by activated, GTP-bound Arf proteins [39]. COPI can also function as a transporter of ER proteins independent of the Golgi [40]. More recently, endosomal coatomer also has been shown to be required for completion of the autophagic process [41]. Indeed, although the COPI complex is not essential for autophagosome formation [42], it plays an important role in the later stages of the autophagic process [41]. All these shared functions between the COP members suggest that depletion of any single member should have, to some extent, the same functional effect. Our data show that depletion of most, but not all (e.g. COPG2), of the COPI subunits results in cell death and an increase in LC3-II positive organelles in different cancer cell lines, pointing to the possibility that the COPI complex might exist in different configurations for its diverse set of functions [43]–[45]. Alternatively, some members of the complex may be less critical for the correct functioning of the COPI complex, perhaps because particular COPI complex members have redundant functions. The essential role of the COP proteins in our studies is in agreement with results found in yeast; with the exception of COPE, yeast COP components are strictly required for viability and inter-compartmental trafficking [46]–[48].

COPB2, first identified as a receptor for activated C-kinase, was found to increase LC3 levels in an siRNA screen for modulators of autophagy [49]. Induction of autophagosomes can result from increased autophagic flux or impaired clearance of the autophagic content [20]. Several lines of evidence in our data led us to hypothesize that the marked increase in autophagosomes and sustained accumulation of LC3-II upon COPI depletion is the result of an impaired autophagic pathway. First, knockdown of COPI members stabilized or allowed the accumulation of p62 in multiple cancer cell lines. Second, inhibition of autophagic flux using a chemical inhibitor together with COPB2 knockdown did not further increase the levels of the lipidated form of LC3. Third, colocalization experiments with tfLC3 revealed defective autolysosome maturation. Finally, recycling of core components critical for the autolysosomes appears to be impaired. These results suggest that depletion of COPI members efficiently promotes abortive autophagy. Our data are consistent with two previous reports [41], [50], both showing that knockdown of COPB2 leads to the accumulation of autophagosomes owing to decreased lysosomal processing. Our results together with these of previous studies show that most, but not all, COP subunits are required for completion of the autophagic process. Remarkably, depletion of COPB2 generated doughnut-like shapes, which are LAMP2-positive, suggesting that while autophagosomes still fuse with lysosomes, the lysosomal dissociation is impaired. Another possibility is that the COPI proteins are required for the pH changes needed for the degradation of the autophagosomal content, which would be in agreement with the observation that COPB1 is involved in the pH-dependent formation of transport vesicles [51].

Since COPI proteins are involved in cargo-regulating transport between the Golgi and ER, depletion of this complex may result in the accumulation of proteins in the ER, leading to ER stress accompanied by activation of the UPR. Although the UPR is generally viewed as a cytoprotective response to protect the ER and to limit cellular damage by the accumulation of unfolded or misfolded proteins, prolonged ER stress can result in cell death [25], similar to prolonged or non-productive autophagy. Indeed, disturbance of ER homeostasis that cannot be rescued by the UPR results in autophagy and cell death. In the case of COPI depletion, however, we found that abortive autophagy (either by chemical inhibition or by siCOPB2) preceded ER stress and is required for the induction of ER stress. Although several reports have proposed that autophagy is downstream of ER stress [52], our results together with another report [53] suggest that autophagy may regulate the UPR pathway and cause ER stress. Targeting cellular components to aggravate ER stress could be a therapeutically attractive strategy, because ER stress is modestly activated in many cancers, particularly breast cancer, compared with normal tissue [54], [55]. This type of therapeutic targeting might be applied in both apoptosis-resistant and apoptosis-sensitive cancer cells. Indeed, this idea is supported by our findings in MDA-MB-231 breast cancer cells, which are competent to undergo apoptosis and do undergo death through abortive autophagy upon COPI depletion. Until now it was unclear whether cancer cells with intact apoptotic machinery would enter autophagy as a death mechanism. Although our data emphasize a unique role for COPI in the induction of ER stress, this observation is in contrast with previous results [41] showing a lack of ER stress after COPI depletion. The differences may result from the use of cancer cells instead of normal cells to investigate ER stress. Indeed, we used cancer cell lines, which have higher levels of basal ER stress and thus are more prone to the further elevation of ER stress to levels inducing cell death. Further, many cancer cell lines have defects in the apoptotic pathway potentially allowing autophagic and ER stress induced cell death to be manifest. This sensitivity of cancer cell lines to ER stress may further be exploited as a therapeutic approach. The COPI complex may fine tune the delicate balance of ER stress in cancer cells and may therefore explain the higher levels the COPI complex members in cancer cells and tissues than in normal cells.

While the function of COPI in cellular trafficking is well established, its effect on downstream signaling is not fully elucidated. A protein array performed on cells with downregulation of COPB2 at different time points revealed an effect of COPI on mTOR function, as analyzed by phosphorylation of its downstream effector 4-EBP. Indeed, a potential important role for COPI in TORC1 regulation is supported by a recent study showing COPI as a key regulator of the mTORC1-S6K signaling pathway [56]. In addition, intracellular trafficking by Rabs and Arf GTPases has been shown to be important for mTORC1 activation, possibly through regulation of the subcellular localization of mTORC1 [57].

Although much information is available about the function of the COPI complex, its relevance to cancer has been underexplored. To better understand its role in cancer, we analyzed several publicly available databases and found that COP proteins are frequently overexpressed in different types of cancer. Our results indeed show that high expression of COPI proteins correlated with a lower survival rate, indicating a role for COPI in cancer. In addition, COPI subunits were also higher expressed in a set of breast cancer cell lines compared to a MCF10A, a non-transformed breast cell line. In contrast to MDA-MB-231 breast cancer cells in which COPB2 depletion induced cell death, abortive autophagy and ER stress, COPB2 knock down in MCF10A cells had modest effect on viability, induced autophagic flux and did not increase ER stress (Fig, S9B–D), indicating a therapeutic opportunity if inhibitors targeting one or more of the components of COPI can be developed. Indeed, both autophagy and ER stress responses represent adaptive mechanisms for the survival of cancer cells [27]. Aggravating those processes by e.g. decreasing the expression of COPI, may convert their protective effect into a cell death mechanism. Recently, COPA emerged as a target in mesothelioma [58], therefore identifying COP proteins as potential therapeutic targets for cancer. A meta-analysis also identified COPB2 as one of four downstream targets of PKCι [59], which we have shown to contribute to poor prognosis through loss of apical-basal polarity and overexpression of cyclin E in ovarian cancer [60]. Whether COP proteins are associated with a worse outcome for cancer patients due to their effects on cell polarity or on autophagy modulation, is a subject for future investigation.

In summary, targeting productive autophagy in cancer cells and converting the process into a cell death mechanism is a promising therapeutic avenue, particularly for cancer cells in which apoptosis is compromised. Further research is needed to validate whether the induction of abortive autophagy will specifically target cancer cells, without causing toxic effects on normal cells. Regardless, here we have shown that targeting the COPI complex is a potent mechanism to block productive autophagy and stimulate cell death in cancer cells.

## Supporting Information

Figure S1 **Screen for modulators of autophagy in cancer cells stably expressing GFP-LC3.** The Accell library targeting 71 genes was transfected in (A) MDA-MB-231, (B) U2OS, and (C) SKOV3 cancer cells. Appropriate positive and negative controls for the small library screen were used. GFP-LC3 punctate formation was analyzed with an IN Cell Analyzer 1000 Cellular Imaging and Analysis system. Genes were sorted based on the percentage of cells with more than 10 punctate dots (% autophagy).
(TIF)

Figure S2 **Validation of efficient knockdown by siRNA treatment.** MDA-MB-231 breast cancer cells were incubated with the indicated siRNAs for 72 h and analyzed for knockdown of the corresponding protein. *, aspecific band.
(TIF)

Figure S3 **Knockdown of COPB2 increases Annexin V-positive MDA-MB-231 breast cancer cells.** MDA-MB-231 cells were treated with siRNA against COPB2 and Annexin V staining was compared to control cells transfected with risc free (RF). Scale bar: 50 µM.
(TIF)

Figure S4 **COPB2 depletion impairs autophagosomal and lysosomal recycling.** (A) GFP-LC3 transfected MDA-MB-231 cells treated with the indicated siRNAs were assessed for organelle mean area using the IN Cell Analyzer 1000 Cellular Imaging and Analysis system. Results shown are mean ± SD triplicates of two independent experiments. ***, p<0.001. (B,C) Transmission electron microscopy analysis of MDA-MB-231 cells after knockdown of COPB2 compared to cells starved from serum and glucose (SF/GF). White arrowheads indicate autophagosomes. Scale bar indicates 2 µm. (D) MDA-MB-231 cells transfected with RF or siRNA targeting COPB2 were analyzed by fluorescence microscopy for LAMP2 (green), COPB2 (red) and nucleus (blue) 72 h post transfection.
(TIF)

Figure S5 **COPB2 depletion blocks rapamycin-induced autophagy.** U2OS cancer cells were treated 48 h with COPB2 siRNA prior to rapamycin (rapa) treatment (100 nM, 24 h) or BafA1 treatment (50 nM, 24 h). Lysates were analyzed for the indicated proteins.
(TIF)

Figure S6 **COPI depletion inhibits complete degradation of autophagosomes in U2OS cells.** tfLC3-expressing U2OS cells were treated with control siRNA (RF) or COPB2 siRNA for 72 h and BafA1 (50 nM) or rapamycin (100 nM) for 24 h. Colocalization of GFP and mRFP was assessed by confocal microscopy. Arrow, RFP-positive/GFP-positive puncta (autophagosome); arrow with open arrowhead: RFP-positive/GFP-negative puncta (autolysosome). Scale bar first column: 50 µM; scale bar last column: 25 µM.
(TIF)

Figure S7 **Abortive autophagy induced by Bafilomycin A1 decreases AKT activity in multiple breast cancer cell lines.** Indicated cell lines incubated for 24 h with BafA1 (50 nM) were analyzed for expression levels of LC3, and total and phosphorylated (pS473AKT, pT308AKT) AKT levels.
(TIF)

Figure S8 **COPB2 is overexpressed in serous ovarian cancer.** COPB2 levels in serous cancer tissue compared with benign tissue in the UCSF dataset.
(TIF)

Figure S9 **Differential expression and function of COPI in cancer cells versus normal cells.** (A) Protein levels of COPI members in different breast cancer cell lines were compared to MCF10A. (B) Cell number of COPB2 depleted MCF10A cells was compared with control cells (Ctr) or cells treated with transfection reagent (mock) or non-targeting siRNA (RF) for 72 h. Results shown are mean ±SD of four replicates. *, p<0.05. (C) Validation of efficient knockdown of COPB2 in MCF10A cells after 72 h of siRNA treatment. (D) Using western blot analysis, MCF10A cells were analyzed for the indicated proteins after knock down of COPB2 for 72 h.
(TIF)

Table S1 **List of positive hits from the siRNA library screening of the human kinome for modulators of viability in cancer cell lines of different lineages.**
(XLS)

Table S2 **List of Accell siRNA pools used to screen for autophagy modulators in cancer cell lines of different lineages.**
(XLS)

Table S3 **List of z-scores from the Accell siRNA library screening for modulators of autophagy in cancer cell lines of different lineages.**
(XLS)
