# Supplementary figures and images for: Abortive Autophagy Induces Endoplasmic Reticulum Stress and Cell Death in Cancer Cells
Source: PLoS One. 2012 Jun 26;7(6):e39400. doi: 10.1371/journal.pone.0039400 (PMC3383753; doi:10.1371/journal.pone.0039400)

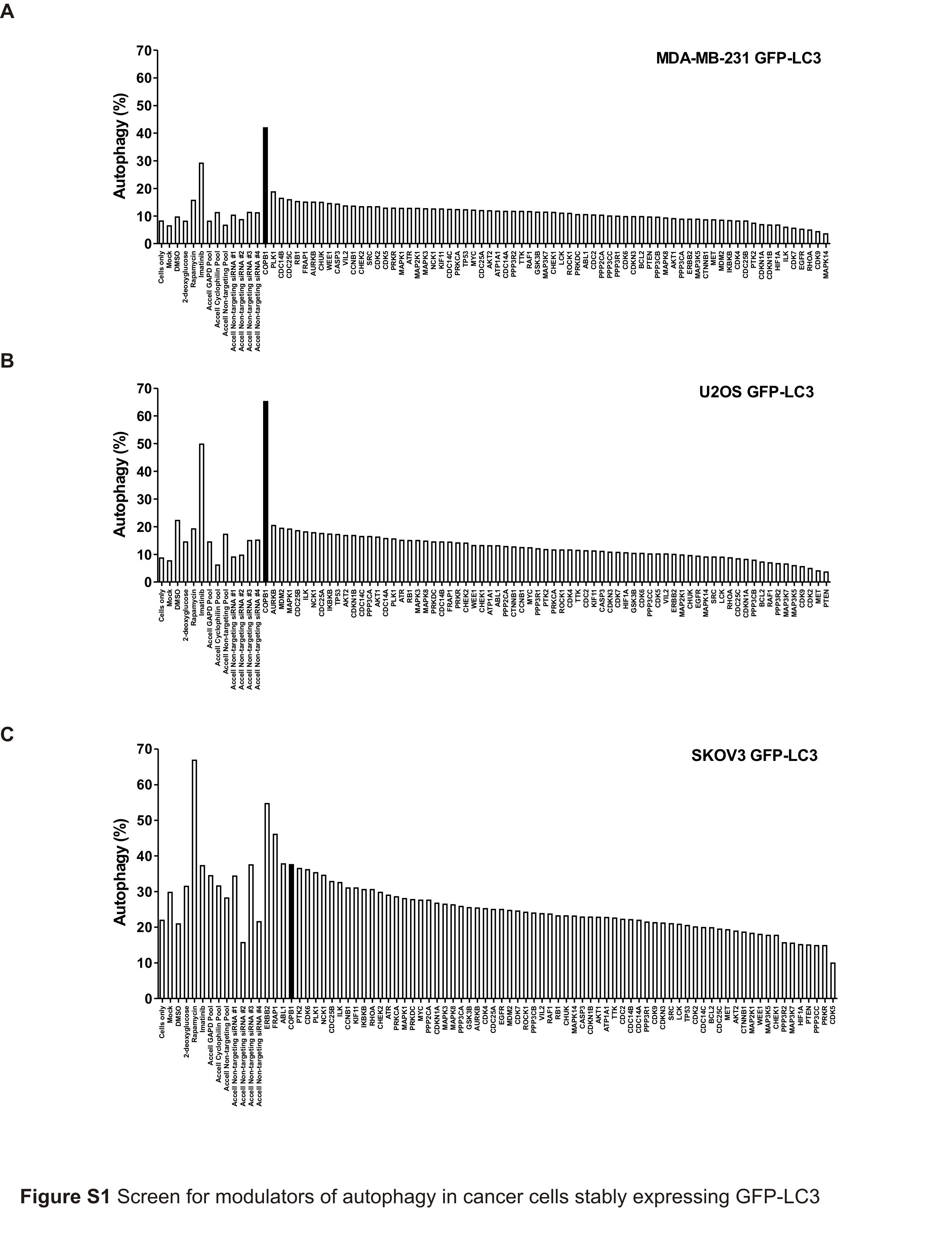

Supplement: Figure S1 — Screen for modulators of autophagy in cancer cells stably expressing GFP-LC3. The Accell library targeting 71 genes was transfected in (A) MDA-MB-231, (B) U2OS, and (C) SKOV3 cancer cells. Appropriate positive and negative controls for the small library screen were used. GFP-LC3 punctate formation was analyzed with an IN Cell Analyzer 1000 Cellular Imaging and Analysis system. Genes were sorted based on the percentage of cells with more than 10 punctate dots (% autophagy). (TIF) [file pone.0039400.s001.tif]

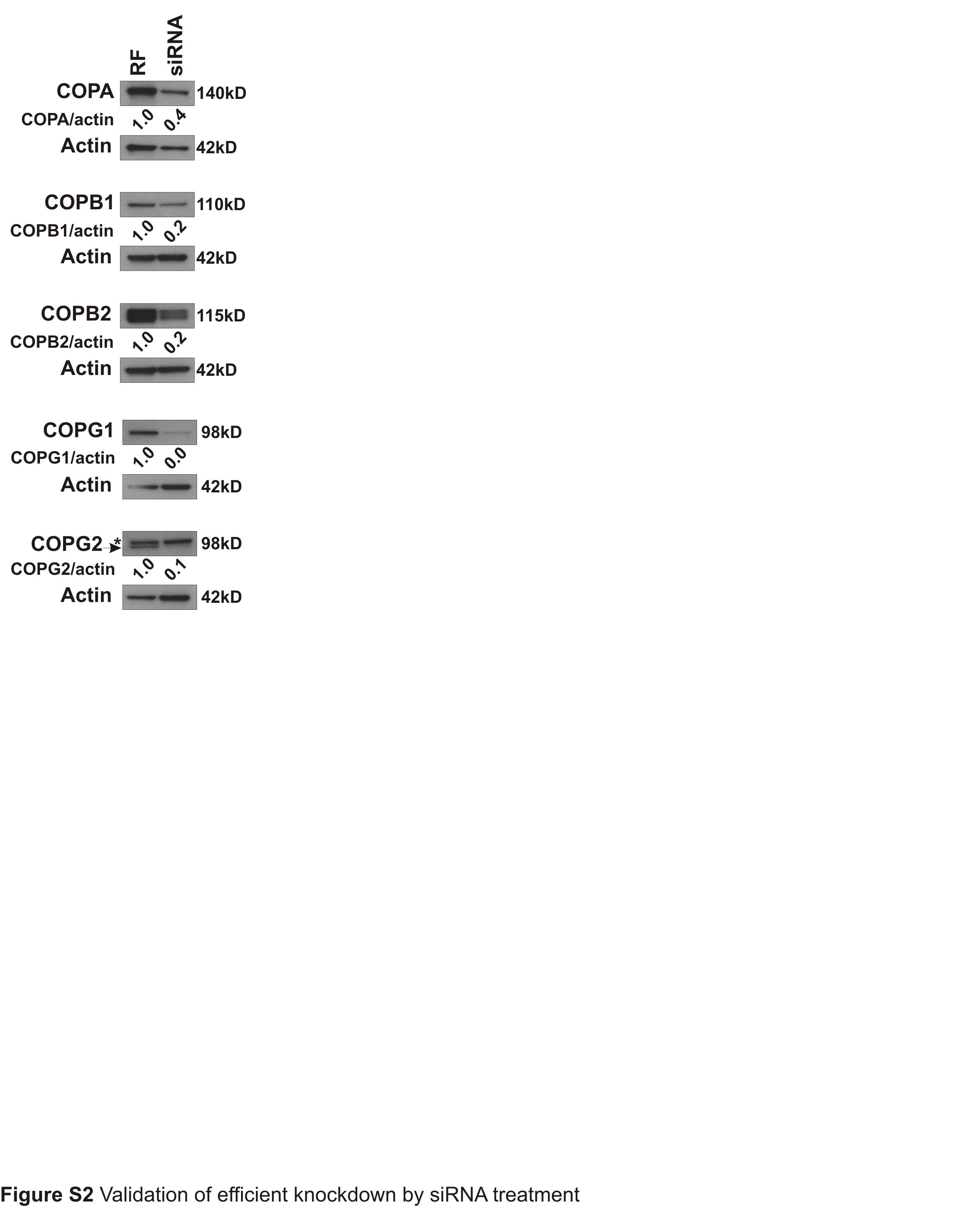

Supplement: Figure S2 — Validation of efficient knockdown by siRNA treatment. MDA-MB-231 breast cancer cells were incubated with the indicated siRNAs for 72 h and analyzed for knockdown of the corresponding protein. *, aspecific band. (TIF) [file pone.0039400.s002.tif]

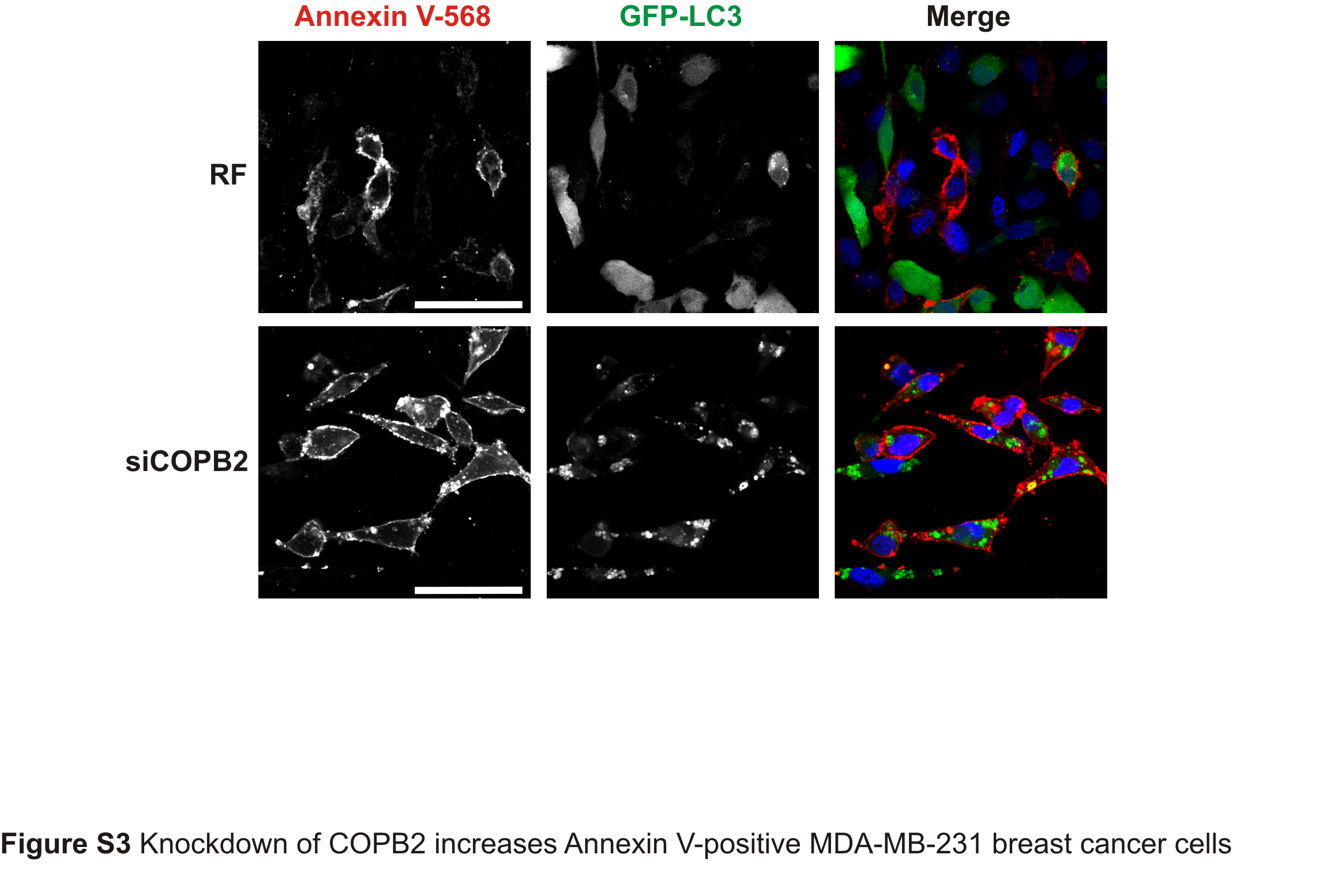

Supplement: Figure S3 — Knockdown of COPB2 increases Annexin V-positive MDA-MB-231 breast cancer cells. MDA-MB-231 cells were treated with siRNA against COPB2 and Annexin V staining was compared to control cells transfected with risc free (RF). Scale bar: 50 µM. (TIF) [file pone.0039400.s003.tif]

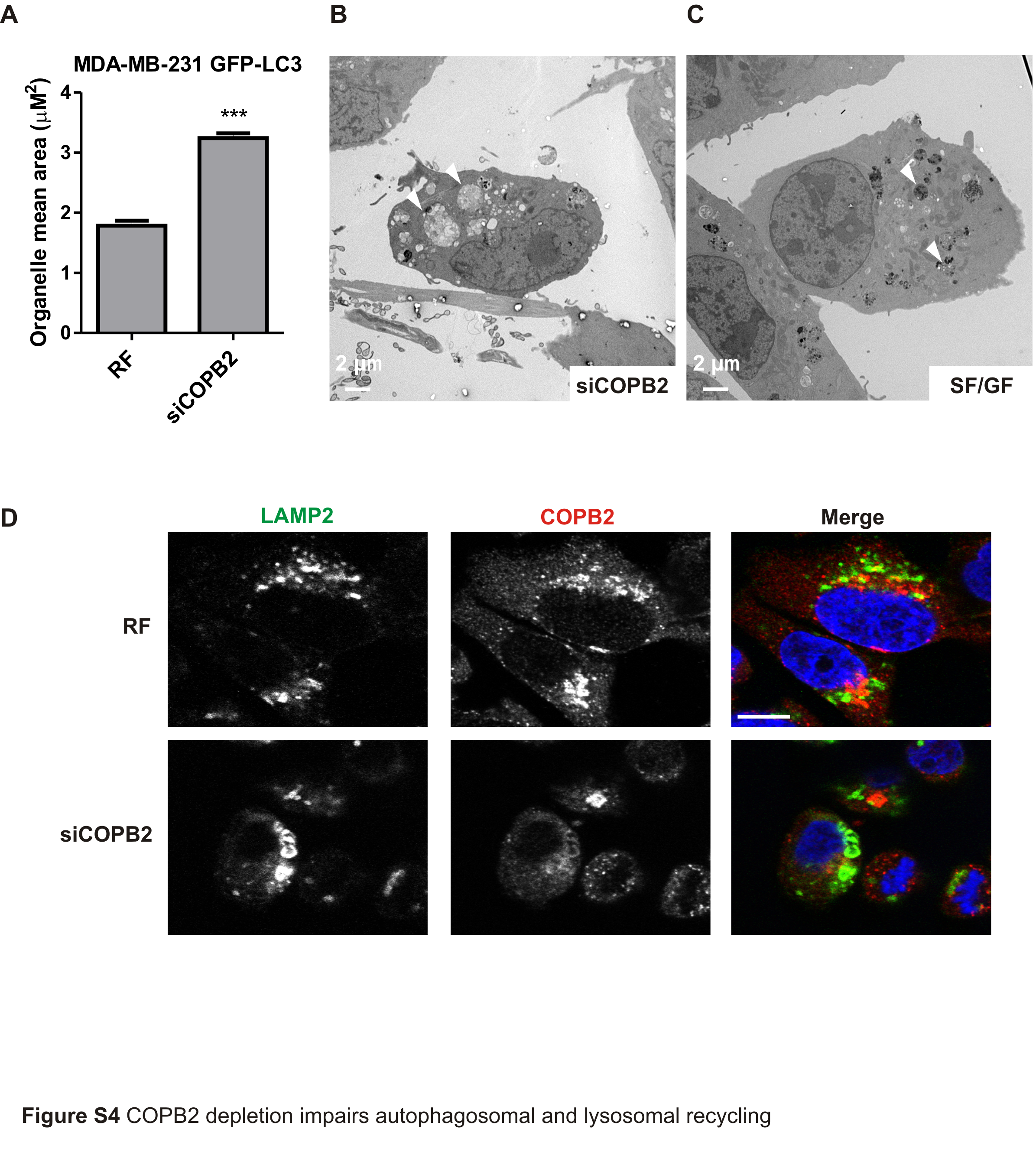

Supplement: Figure S4 — COPB2 depletion impairs autophagosomal and lysosomal recycling. (A) GFP-LC3 transfected MDA-MB-231 cells treated with the indicated siRNAs were assessed for organelle mean area using the IN Cell Analyzer 1000 Cellular Imaging and Analysis system. Results shown are mean ± SD triplicates of two independent experiments. ***, p<0.001. (B,C) Transmission electron microscopy analysis of MDA-MB-231 cells after knockdown of COPB2 compared to cells starved from serum and glucose (SF/GF). White arrowheads indicate autophagosomes. Scale bar indicates 2 µm. (D) MDA-MB-231 cells transfected with RF or siRNA targeting COPB2 were analyzed by fluorescence microscopy for LAMP2 (green), COPB2 (red) and nucleus (blue) 72 h post transfection. (TIF) [file pone.0039400.s004.tif]

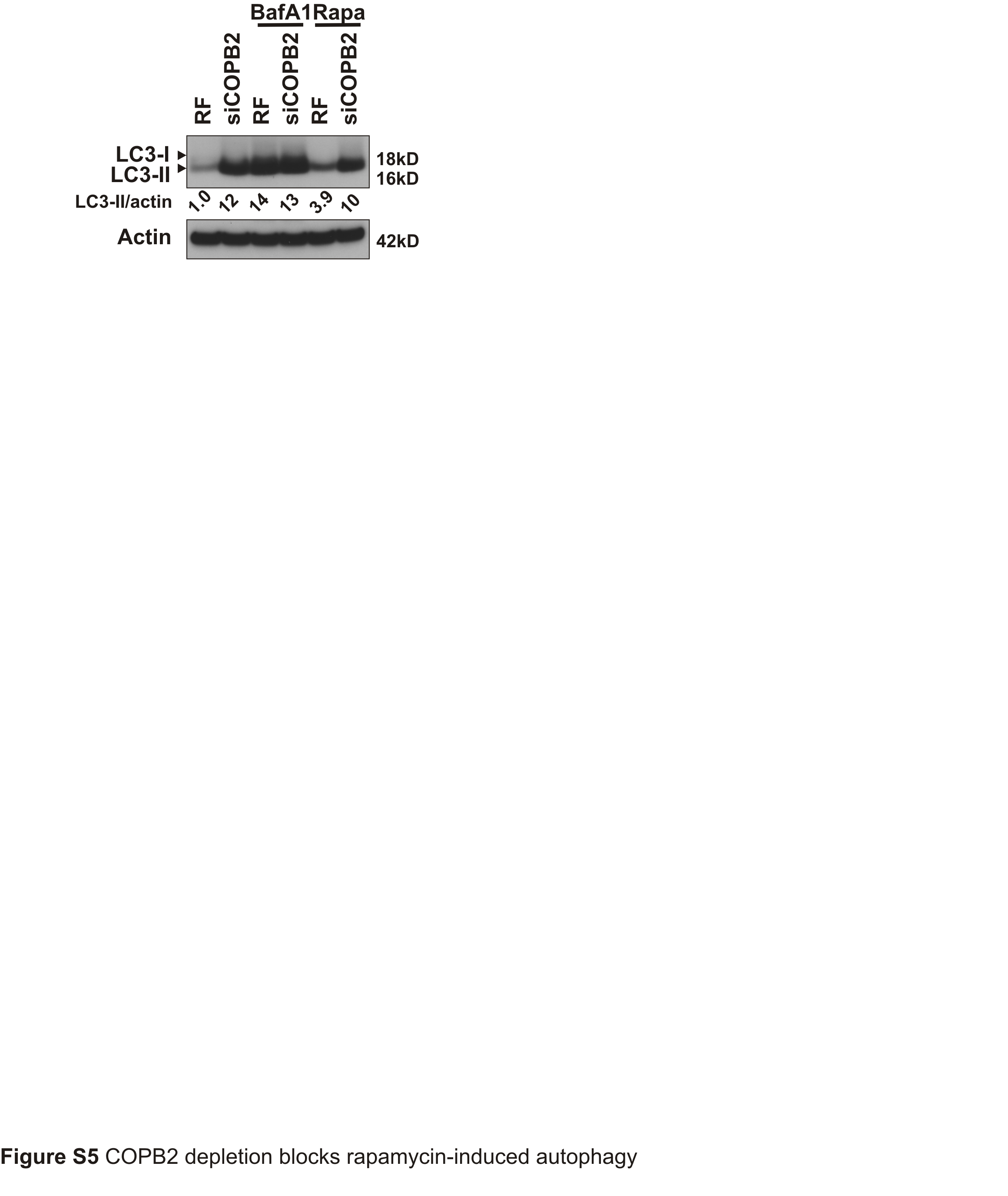

Supplement: Figure S5 — COPB2 depletion blocks rapamycin-induced autophagy. U2OS cancer cells were treated 48 h with COPB2 siRNA prior to rapamycin (rapa) treatment (100 nM, 24 h) or BafA1 treatment (50 nM, 24 h). Lysates were analyzed for the indicated proteins. (TIF) [file pone.0039400.s005.tif]

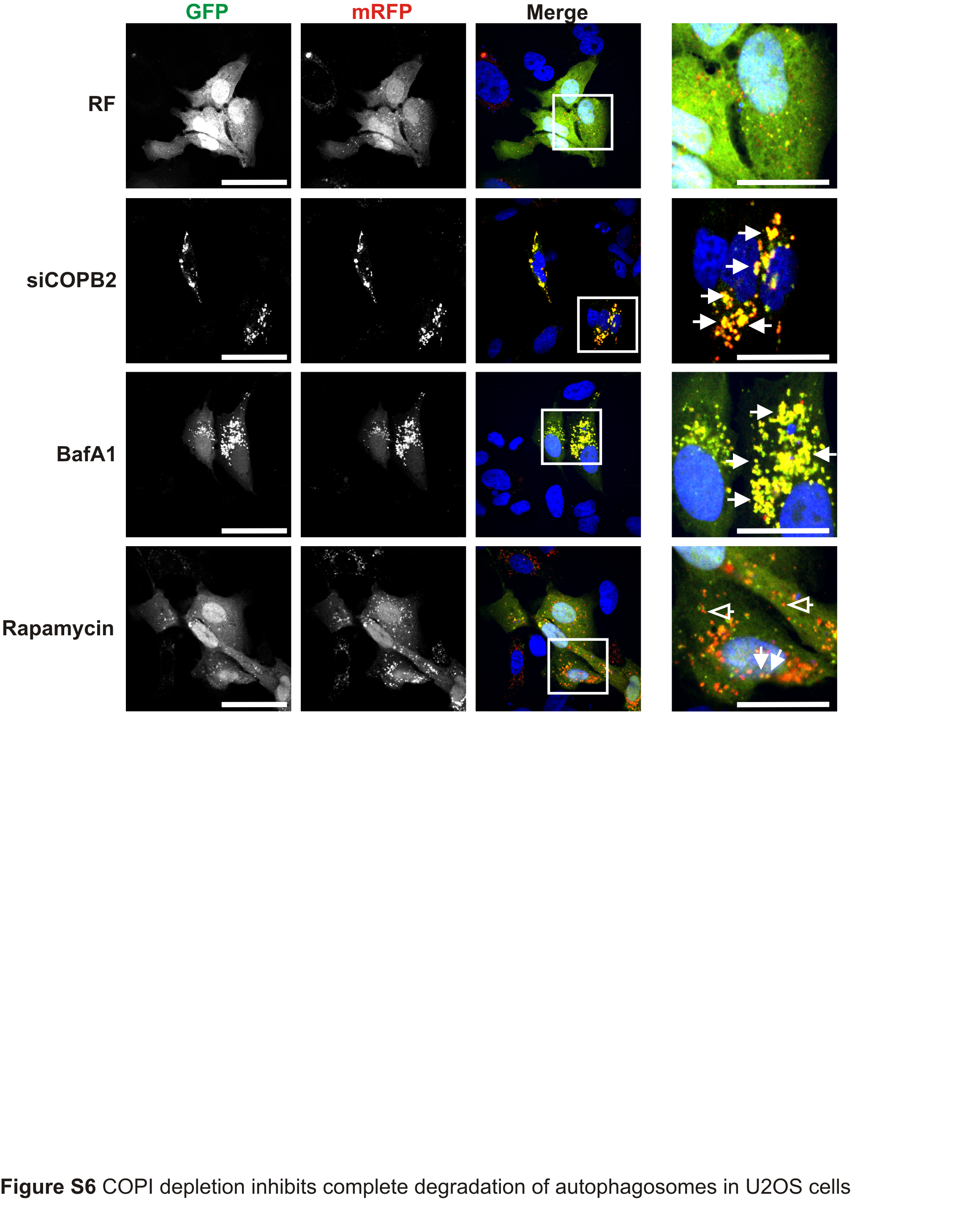

Supplement: Figure S6 — COPI depletion inhibits complete degradation of autophagosomes in U2OS cells. tfLC3-expressing U2OS cells were treated with control siRNA (RF) or COPB2 siRNA for 72 h and BafA1 (50 nM) or rapamycin (100 nM) for 24 h. Colocalization of GFP and mRFP was assessed by confocal microscopy. Arrow, RFP-positive/GFP-positive puncta (autophagosome); arrow with open arrowhead: RFP-positive/GFP-negative puncta (autolysosome). Scale bar first column: 50 µM; scale bar last column: 25 µM. (TIF) [file pone.0039400.s006.tif]

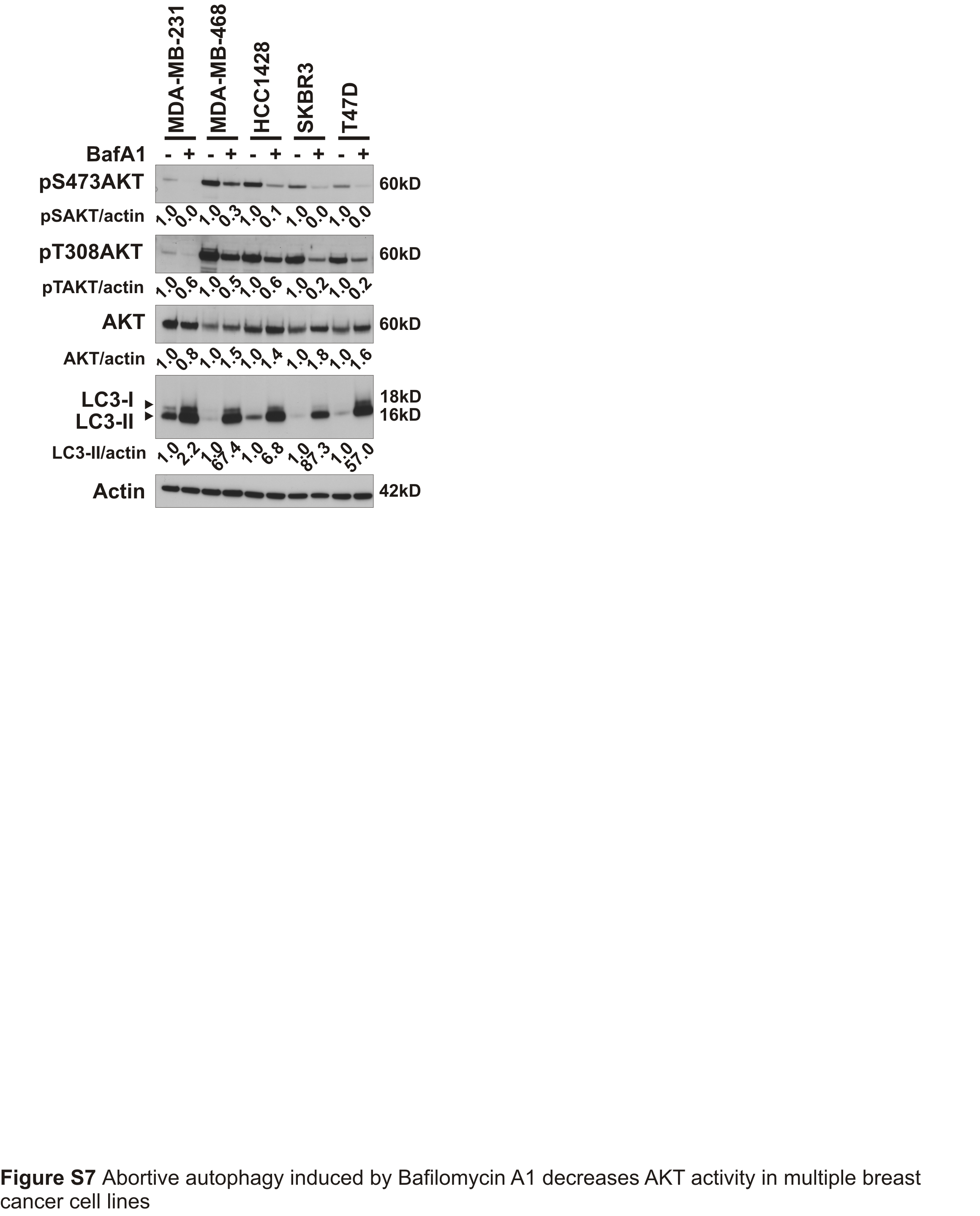

Supplement: Figure S7 — Abortive autophagy induced by Bafilomycin A1 decreases AKT activity in multiple breast cancer cell lines. Indicated cell lines incubated for 24 h with BafA1 (50 nM) were analyzed for expression levels of LC3, and total and phosphorylated (pS473AKT, pT308AKT) AKT levels. (TIF) [file pone.0039400.s007.tif]

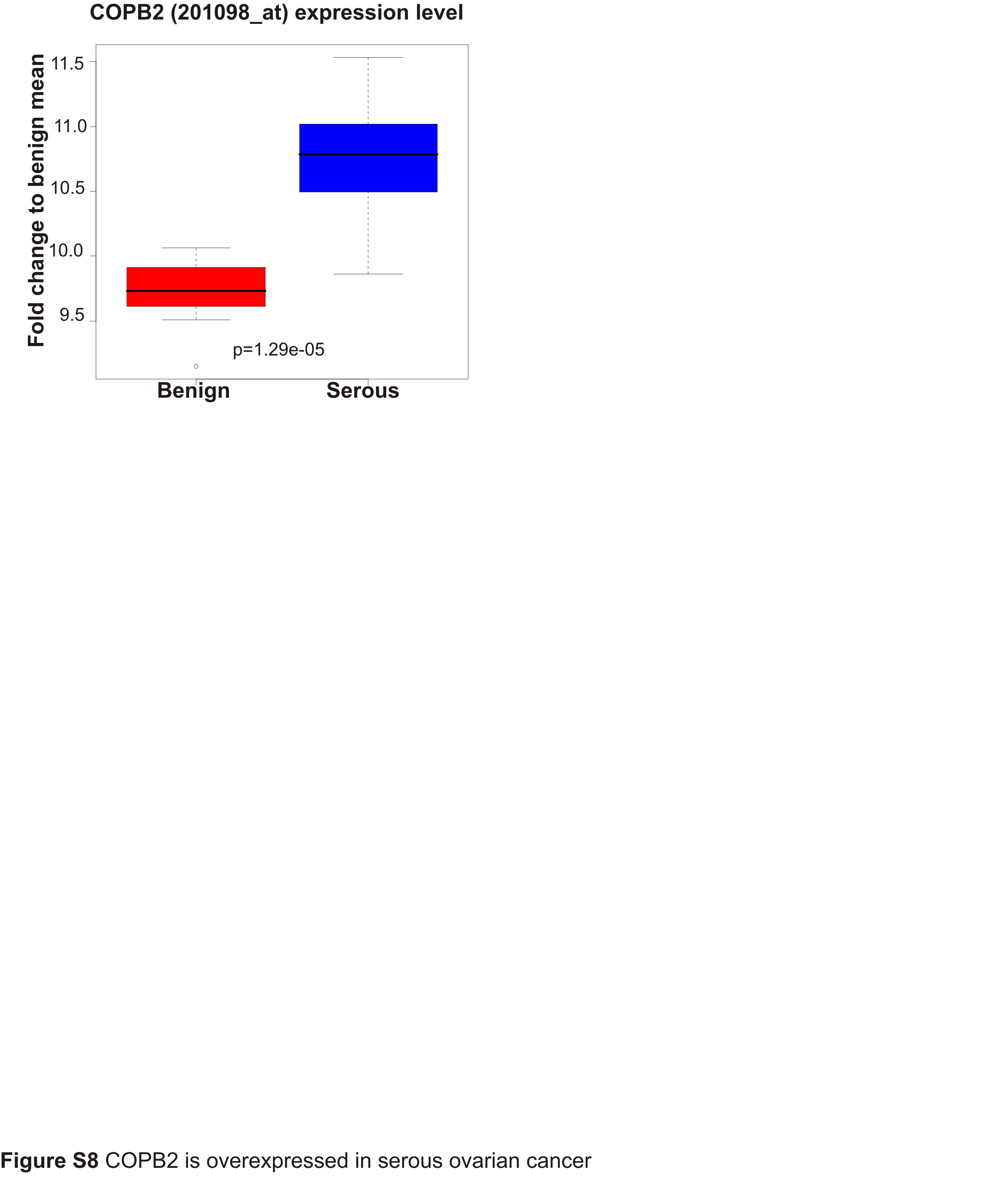

Supplement: Figure S8 — COPB2 is overexpressed in serous ovarian cancer. COPB2 levels in serous cancer tissue compared with benign tissue in the UCSF dataset. (TIF) [file pone.0039400.s008.tif]

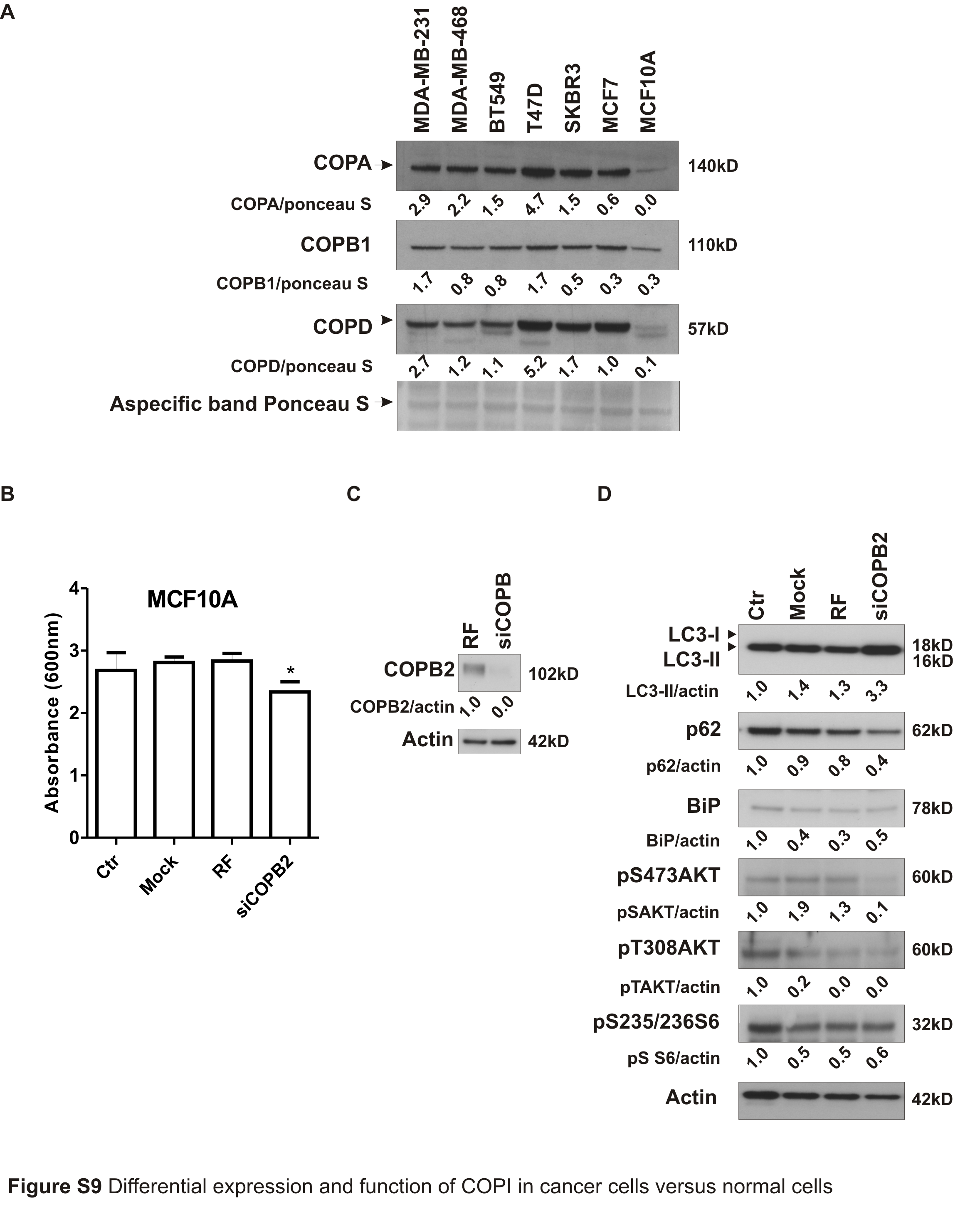

Supplement: Figure S9 — Differential expression and function of COPI in cancer cells versus normal cells. (A) Protein levels of COPI members in different breast cancer cell lines were compared to MCF10A. (B) Cell number of COPB2 depleted MCF10A cells was compared with control cells (Ctr) or cells treated with transfection reagent (mock) or non-targeting siRNA (RF) for 72 h. Results shown are mean ±SD of four replicates. *, p<0.05. (C) Validation of efficient knockdown of COPB2 in MCF10A cells after 72 h of siRNA treatment. (D) Using western blot analysis, MCF10A cells were analyzed for the indicated proteins after knock down of COPB2 for 72 h. (TIF) [file pone.0039400.s009.tif]
